# Eight New Species of *Charinus* Simon, 1892 (Arachnida: Amblypygi: Charinidae) Endemic for the Brazilian Amazon, with Notes on Their Conservational Status

**DOI:** 10.1371/journal.pone.0148277

**Published:** 2016-02-17

**Authors:** Alessandro Ponce de Leão Giupponi, Gustavo Silva de Miranda

**Affiliations:** 1 Programa de Pós-Graduação em Zoologia, Departamento de Invertebrados, Laboratório de Aracnologia, Museu Nacional, Universidade Federal do Rio de Janeiro, Rio de Janeiro-RJ, Brazil; 2 Laboratório de Referência Nacional em Vetores das Riquetsioses, LIRN/CAVAISC-IOC-FIOCRUZ, Rio de Janeiro, RJ, Brazil; 3 Center for Macroecology, Evolution and Climate, Natural History Museum of Denmark (Zoological Museum), University of Copenhagen, Copenhagen, Denmark; CNRS, FRANCE

## Abstract

Eight new species of *Charinus* Simon, 1892 are described for the Brazilian Amazon, from the states of Pará (*C*. *bichuetteae* sp. n., *C*. *bonaldoi* sp. n., *C*. *carajas* sp. n., *C*. *ferreus* sp. n., *C*. *guto* sp. n. and *C*. *orientalis* sp. n.) and Amazonas (*Charinus brescoviti* sp. n. and *C*. *ricardoi* sp. n.). All new species can be differentiated from the other species of the genus by the number of pseudo-articles in basitibia IV, the presence/absence of median eyes, and the shape of the female gonopod. Brazil now becomes the country with the largest diversity of Amblypygi in the world, with 25 known species. Half of the new species described here have a high degree of endangerment: *C*. *bichuetteae* sp. n. is threatened by the flood caused by the hydroelectric dam of Belo Monte, and *C*. *carajas* sp. n., *C*. *ferreus* sp. n. and *C*. *orientalis* sp. n. are endangered by the iron mining in Carajás municipality and surroundings. The *Charinus* species here described are endemic to the Amazon Region, so in order to assure their preservation, it is strongly recommended a special care with their habitats (type localities) which are facing increasing rates of destruction and deforestation.

## Introduction

Amblypygi is a small arachnid order with a worldwide distribution and the largest number of described species in the Neotropics [1, 2]. Currently, *circa* of 170 species are known and several new ones have been described in the last few years from all over the world [3–16]. Despite this increase in knowledge of whip spider diversity, some countries with recognized enormous diversity of fauna still have few known Amblypygi species described. With a 8,514,877 km² territory size [17] and six biomes (Amazon Forest (humid broad leaf forest), Caatinga (small woody and herbaceous deciduous, caducifolious, spiny species [18]), Cerrado (open tree and shrub woodland [19]), Atlantic Forest (costal forest), Pantanal (tropical swampland) and Pampa (grasslands)) Brazil has records of just 17 species of these arachnids in all biomes, except in the Pampa where amblypygids are not found. Its presence in all these heterogenic regions shows Brazil’s potential to harbor high diversity due to proper environmental condition, such as favorable temperature, humidity, and availability of food, each environment in its own way.

Among the different Brazilian biomes, the Amazon region stands out for its huge diversity, enigmatic biogeography, and little knowledge about the species that live there. Influenced by tectonics [20], marine transgression [21–24], climate change [25, 26] paleoenvironmental changes [27], niche conservatism [28–30] and dispersal ability [31], several speciation events occurred in Amazonia during the last 5–3 million years and there is still a lot to be unveiled. Even after centuries of scientific expeditions to that forest, new species and subspecies of big vertebrates, like monkeys [32], continue to be discovered.

Despite of its incredible biodiversity, conservative measures are not efficient and several human activities threaten the Amazonian environment. The current most important are the construction of highways, hydroelectric dams and mining activities. Highways crossing areas of Amazonia effects the dispersal and migration of many species, causes border effect, allows the entry of squatters and causes deforestation by itself [33–35]. Hydroelectric dams kill and force the migration of several species due to floods of large areas; moreover, the reservoir build artificial islands affecting the composition, abundance and richness of the species living on them [36, 37]. Likewise, mining is a central issue, once this practice irreversibly destroy unique habitats, like caves; in Brazil the concern regarding ‘conservation *versus* mining’ is even higher due to recent changes in laws which allows exploitation of protected areas and indigenous lands [38, 39].

With the increasing threats towards the Amazon forest it is important to unveil whip spider diversity before they disappear, as most of them are extremely sensitive to environmental changes [2] and could help identifying priority areas for directing conservation efforts [2, 14]. By now only one species of Charinidae is known from this region [7], but in this paper eight new are described and illustrated; moreover, an identification key is provided for all species from the region. With this, the number of whip spiders' species in the country is almost doubled, and the knowledge of the group morphology is considerably increased.

## Material and Methods

### Ethics statement

All analyzed material was from zoological custodian collections. The name of the collection and their deliberation by the Brazilian government are: Instituto Butantan (**IBSP**; Coleção Entomológica de Aracnídeos e de Miriápodes; process number 02000.004897/2005-99; Deliberation number 147, published in the Official Gazette of the Union (D.O.U.) on 07.28.2006, Section 1, page 100); Museu Nacional do Rio de Janeiro (**MNRJ**; Setor Aracnologia; process number 02000.002444/2002-85; Deliberation number 06, published in the D.O.U. on 10.14.2002, Section 1, page 115); Museu Paraense Emílio Goeldi (MPEG; Coleção Invertebrados; process number 02000.001401/2002-82, Deliberation number 02, published in the D.O.U. on 07.26.2002, Section 1, page 141); Museu de Zoologia da USP (**MZSP**; Coleção de Aracnídeos; process number 02000.001100/2002-59; Deliberation number 02, published in the D.O.U. on 07.26.2002, Section 1, page 14).

### Laboratory procedures

In general, for measurements and nomenclature, we followed the proposals of Quintero [40] and for the nomenclature of the male gonopod we followed Giupponi and Kury [13]. The pedipalp article, called tarsus by Quintero [40], is here divided in distitarsus and tarsal claw (pretarsus) as the two articles are not fused in Charinidae. The measurements of pedipalp articles were taken between the external condiles of each segment in order to establish fixed points and proper length measurements [41]. The measurements were taken from several specimens (number indicated as “n =“) and the median value is given followed by the range in parentheses. The measurement accuracy is indicated in the legend of each figure. The maps were created using ArcMap 10.2 [42], with vector layers for countries and states/provinces and ancillary data including water body data [43].

The following abbreviations are used:

**BR**–Brazil
**FLONA**–Floresta Nacional (National Forest).
**IBSP**–Instituto Butantan, São Paulo, Brazil;
**LoL1** –*Lobus lateralis primus*;
**LoL2** –*Lobus lateralis secundus*;
**MNRJ**- Museu Nacional do Rio de Janeiro, Rio de Janeiro, Brazil;
**MPEG**–Museu Paraense Emiliano Goeldi, Belém, Brazil;
**MZSP**–Museu de Zoologia da Universidade de São Paulo, São Paulo, Brazil;
**PA**–Pará;
**PI**–*Processus Internus*;
**SP**–São Paulo;
**UFSCar**–Universidade Federal de São Carlos, São Paulo, Brazil;

### Nomenclatural acts

The electronic edition of this article conforms to the requirements from the amended International Code of Zoological Nomenclature, and hence the new names contained here are available under that Code from the electronic edition of this article. This published work and the nomenclatural acts it contains have been registered in ZooBank, the online registration system for the ICZN. The ZooBank LSIDs (Life Science Identifiers) can be resolved and the associated information viewed through any standard web browser by appending the LSID to the prefix "http://zoobank.org/". The LSID for this publication is: urn:lsid:zoobank.org:pub:876ED555-65EC-467D-9B1A-666E01AC5F3B. The electronic edition of this work was published in a journal with an ISSN, and has been archived and is available from the following digital repositories: PubMed Central, LOCKSS.

### Additional material examined

*Charinus vulgaris* Miranda & Giupponi, 2011, Female holotype: BRAZIL: Rondônia: Porto Velho, Bairros: São João Bosco, Rio Madeira and Santo Antônio, II-2011, Miranda, G. S. leg. (MNRJ 09106); *Charinus bromeliaea* Jocque & Giupponi, 2012, Female holotype (MNRJ 09185), French Guyana, Savanna Roche La Virginie, (4°11'24.00"N, 52°08'60.00"W), collected on 20 august 2008 by M. Jocque in *Achmea* cfr. *melionii* bromeliads.

## Results

### Taxonomic treatment

**Fig 1:**
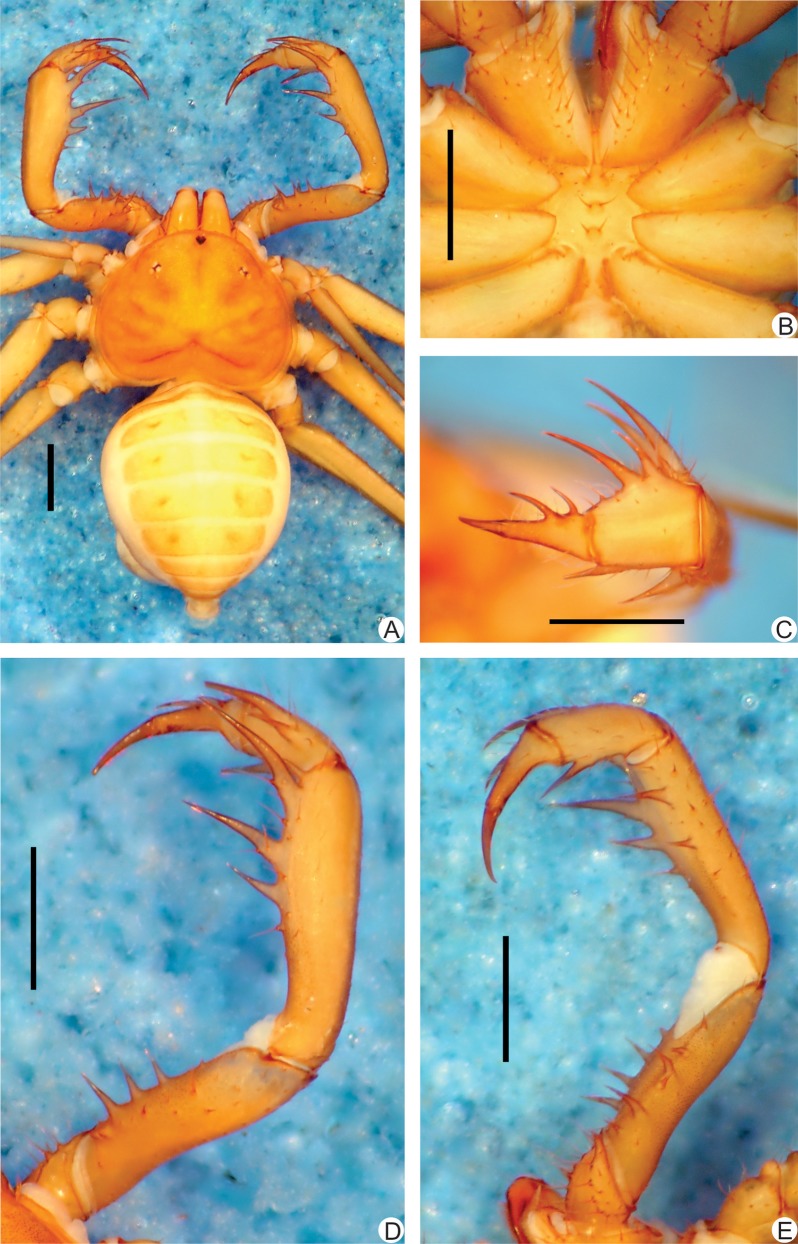
**A-E.** Habitus and details of the sternum and pedipalp of *Charinus brescoviti* sp. n. (female, MNRJ 9186). **A.** Dorsal habitus; **B.** Sternum; **C.** Frontal view of left the pedipalp basitiba, distitiba and claw; **D.** Dorsal view of the right pedipalp; **E.** Ventral view of the left pedipalp. Scale bars: 1mm.

**Fig 2:**
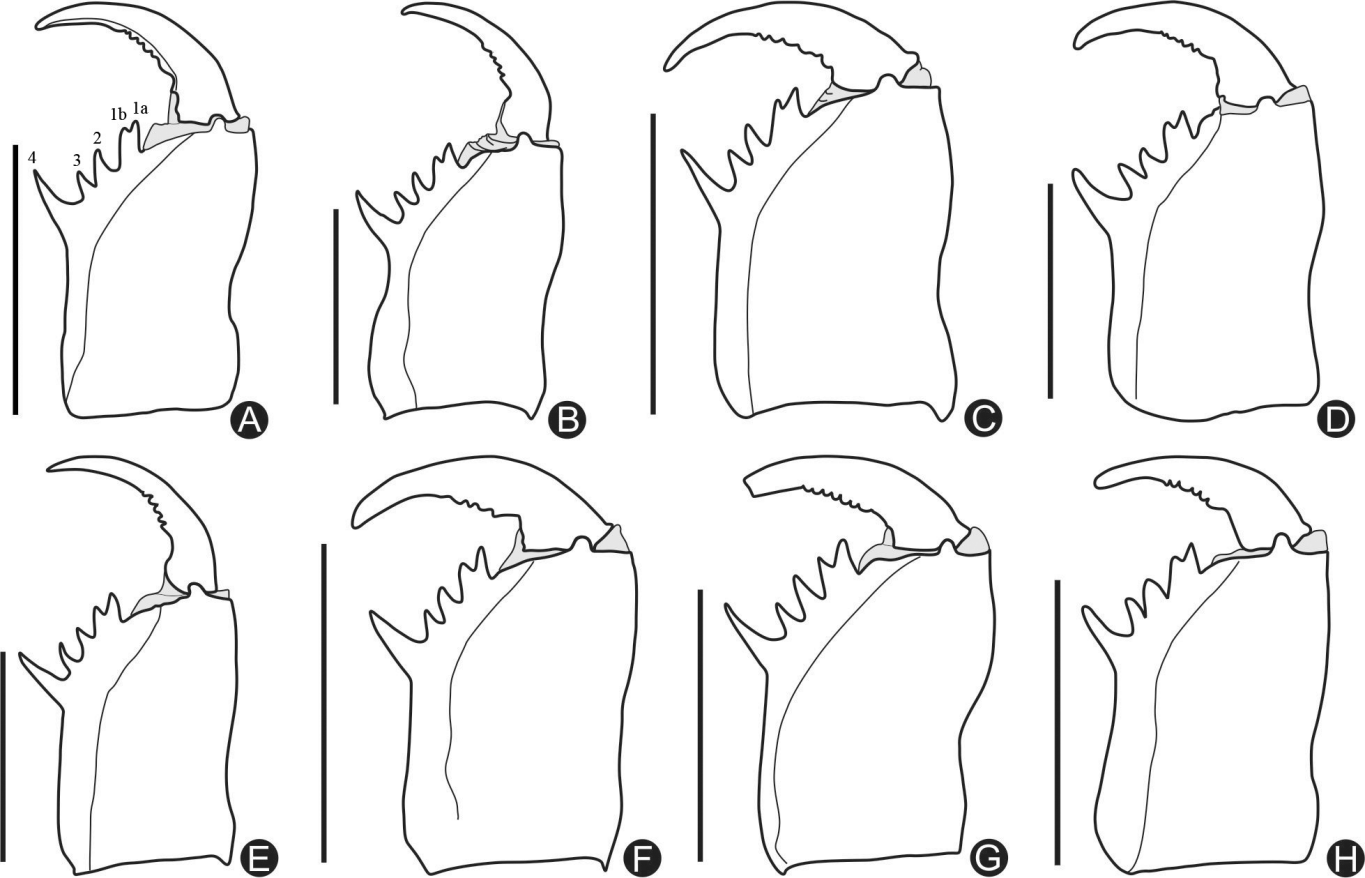
**A-H.** Mesal view of the right chelicerae of all new species here described. **A.** *Charinus brescoviti* sp. n. (female paratype, IBSP 149); **B.** *Charinus ricardoi* sp. n. (female paratype, MZSP 22036); **C.** *Charinus bonaldoi* sp. n. (female paratype, MPEG 0061); **D.** *Charinus guto* sp. n. (female paratype, MZSP 41460); **E.** *Charinus carajas* sp. n. (male paratype, MZSP 28291); **F.** *Charinus orientalis* sp. n. (female paratype, MZSP 29118); **G.** *Charinus ferreus* sp. n. (female paratype, MZSP 29106); **H.** *Charinus bichuetteae* sp. n. (male paratype, MNRJ 9173). Scale bars: 1mm.

**Fig 3:**
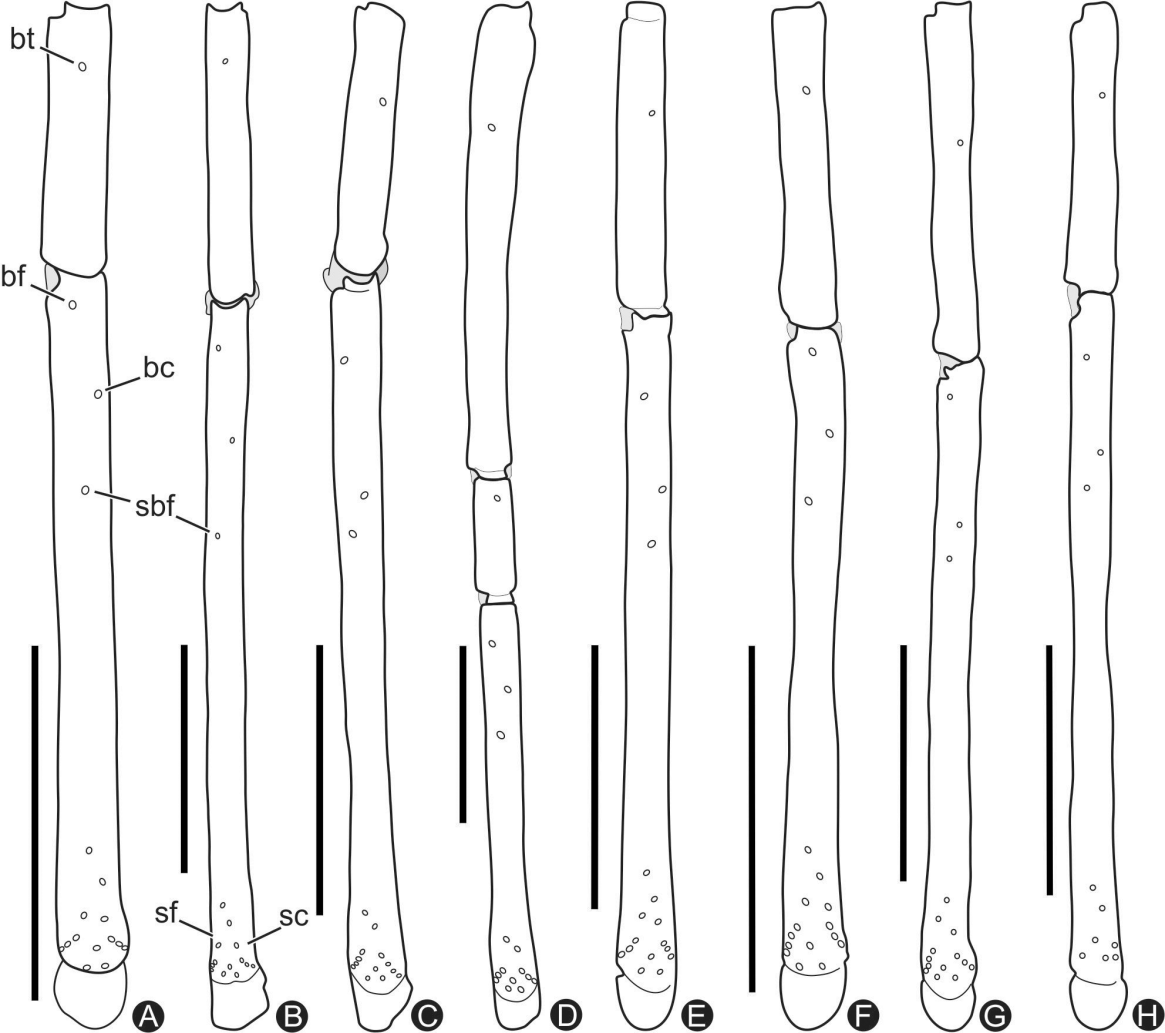
**A-H.** Trichobothria of left basitibia IV and distitibia IV. **A.** *Charinus brescoviti* sp. n. (female holotype, IBSP 149); **B.** *Charinus ricardoi* sp. n. (female holotype, MZSP 22036); **C.** *Charinus bonaldoi* sp. n. (female paratype, MPEG 0061); **D.** *Charinus guto* sp. n. (female holotype, MZSP 48146); **E.** *Charinus carajas* sp. n. (female holotype, MZSP 29136); **F.** *Charinus orientalis* sp. n. (female paratype, MZSP 29118); **G.** *Charinus ferreus* sp. n. (female holotype, MZSP 29104); **H.** *Charinus bichuetteae* sp. n. (male paratype, MNRJ 09173). Acronyms: *bt*, basotibial; *bf*, basofrontal; *bc*, basocaudal; *sbf*, sub-basofrontal; *sc*, caudal series; *sf*, frontal series;. Scale bars: 1mm.

**Fig 4:**
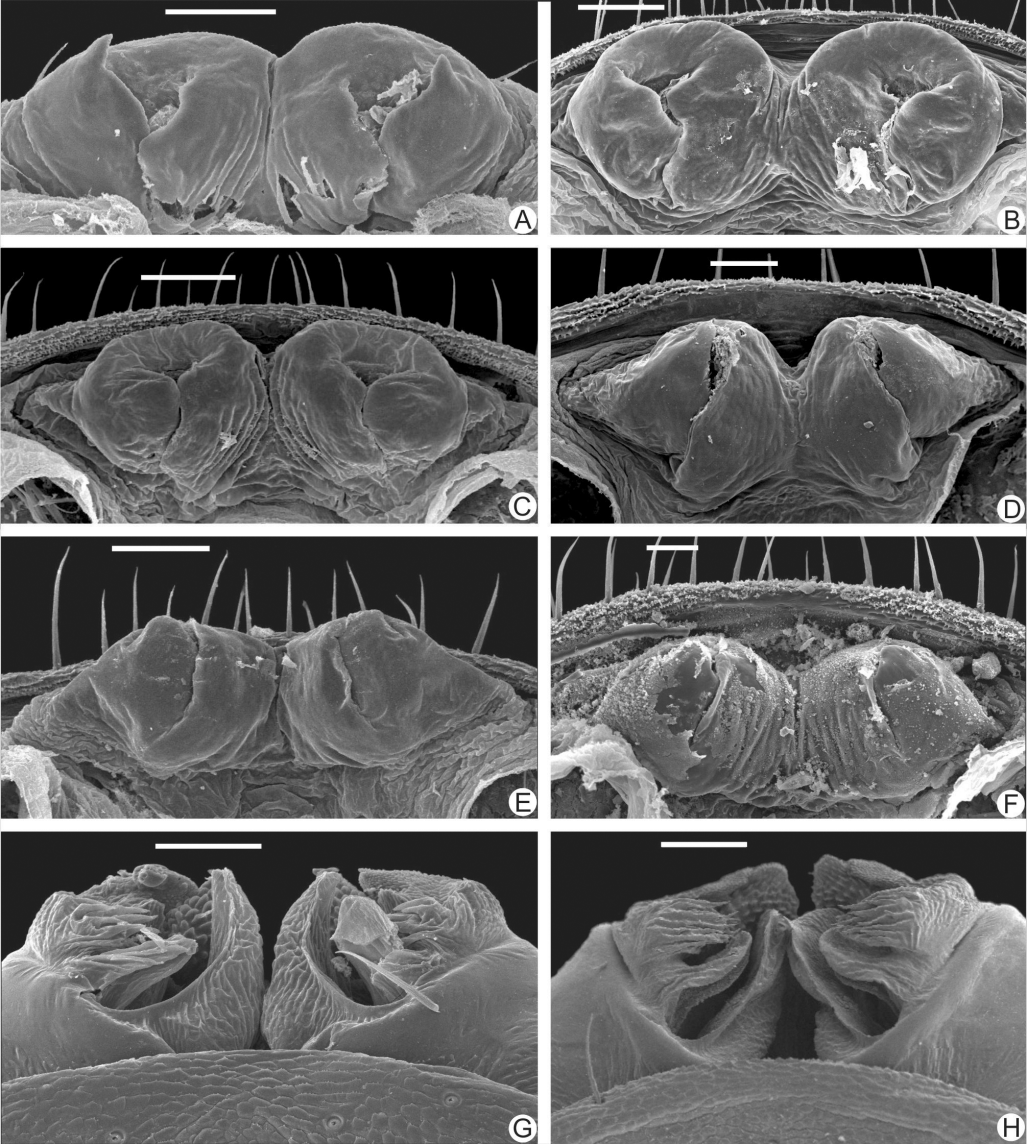
**A-H.** Female and male gonopods of the new species described in this paper. **A-F.** Female gonopods. **A.** *Charinus brescoviti* sp. n. (IBSP 149); **B.** *Charinus ricardoi* sp. n. (MZSP 22036); **C.** *Charinus bonaldoi* sp. n. (MPEG 061); **D.** *Charinus guto* sp. n. (MZSP 48146); **E.** *Charinus carajas* sp. n. (MZSP 29132); **F.** *Charinus orientalis* sp. n. (MNRJ 09249). **G-H.** Male gonopods. **G.** *Charinus carajas* sp. n. (MZSP 29121); **H.** *Charinus ferreus* sp. n. (MZSP 29106). Scale bars of figures. A, B, C, E, G, H: 100μm. Scale bars of figures. D, F: 50μm.

***Charinus brescoviti* new species.** urn:lsid:zoobank.org:act: ED0B1B23-2274-449F-B9EE-EDAE43A48902

(Figs 1A–1E, 2A, 3A and 4A)

#### Etymology

This species is named after Dr. Antônio Domingos Brescovit (Instituto Butantan, SP, BR), in recognition of his contribution to arachnology.

#### Type material

**Holotype:** BRAZIL: *Amazonas*: Araçá River, Piaçaba, 18.v.1982, B. Mascarenhas (Female, IBSP 149). **Paratypes:** BRAZIL: *Amazonas*: Araçá River, Piaçaba, 18.v.1982, B. Macharenhas (1 female, IBSP 149; 1 female and 1 juvenile, MNRJ 9186).

#### Diagnosis

Well-developed median and lateral eyes; small and rounded meta and mesosternum; large basal spine pedipalp distitarsus (2/3 the distal length); basitibia of leg IV divided in two pseudo articles; trichobothria of basitibia IV (*bt*) on the proximal third of the article; distitibia IV with 16 trichobothriae, equidistant basal trichobothria (*bf*, *bc* and *sbf*); brownish-yellow body color; cushion-like gonopods with small lateral projections directed backwards and internal seminal receptacles.

#### Description

**Carapace** (Fig 1A): flattened, wider than longer (ratio approximately 4/5). Prominent lateral and median eyes; from the median eyes starts a thin median furrow that reaches around the median area of the lateral hump pair, behind the lateral eye spots. Anterior margin with 6 small setae. Corners of anterior margin extending downwards in a wide, roundish boss. Many tiny punctuations, more abundant in the frontal area. Three pairs of deep furrows and a deep, oval fovea. First pair of furrows placed just behind the lateral boss and not reaching the middle line. Lateral eyes without cornea and clearly defined lens (only a small roundish knob). Frontal process well developed, much longer than wider, with blunt, reborded apex.

#### Sternum (Fig 1B)

Tri-segmented, all pieces weakly sclerotized. Tritosternum with a round basis and projected anteriorly in a small blunt tubercle, which reaches the base of the chelicerae, with 2 apical, 2 median and 2 basal setae. Middle piece rounded, convex, with 2 setae. Third piece rounded and convex, subequal to the middle piece, with two setae. Sternites separated from each other by the diameter of the middle piece.

#### Abdomen (Fig 1A)

Oblong, with almost indistinguishable punctuations, finer than that on the carapace.

#### Chelicera (Fig 2A)

Cheliceral furrow with 4 internal teeth; first tooth bifid, Ia slightly bigger than Ib. Second and third teeth subequal. Fourth tooth twice as long as the others and stouter. Teeth length: IV>Ia>Ib = II>III. Claw with 6 denticles, the basal larger.

#### Pedipalp

**Trochanter** (Fig 1D and 1E): large ventral apophysis on the posterior border of the article, spiniform, bearing seven large setae, and with a blunt tip pointed forwards; two subequal spines, one at the median third and the other at the distal tip of the prolateral face. **Femur** (Fig 1D and 1E): 3–4 dorsal spines (I>II>III>IV) with two prominent setiferous tubercle before the first spine; 4 ventral spines (main series) with one small accessory spine before the first, this the same size of spine IV (I>II>III>IV). **Tibia** (Fig 1D and 1E): dorsal main series with three spines (I>II>III); small accessory spine before the first spine; spine II two thirds spine I and spine III one third the size spine I. Spine I with a setiferous tubercle on its first third. 2 ventral spines, the proximal two thirds the distal. **Basitarsus** (Fig 1C, 1D and 1E): 2 dorsal spines, the basal 2/3 the distal. 1 ventral spine at the distal half, subequal to the dorsal basal spine. **Distitarsus** (Fig 1C): with 2 large curved spines, the distal half the size of the article, and the basal half distal spine. Cleaning organ about ½ the article length. **Claw** (Fig 1C): long, with an acute, curved tip.

#### Legs

All setose. Ventral corner of the prolateral face of femora II-IV projecting in a distinct spiniform process. **Femur length** I>III>IV>II. Tibia I with 23 articles. Tarsus (basitarsus+distitarsus) I with 37–39 articles. **Leg IV**: **Basitibia:** divided into 2 pseudo-articles, with one trichobothrium on both pseudo-segments; the trichobothrium of the proximal pseudo-article is between the median and the distal third; the trichobothrium of the distal pseudo-article is on the basal third. **Distitibia** (Fig 3A): 3 basal and 13 distal trichobothria (total of 16); trichobothrium *bc* half way to *bf* and *sbf*. **Basitibia-distitibia length** DT>BT1>BT2.

#### Measurements

**Female** (n = 3): Cephalothorax: Length: 2.1 mm (2.0–2.3), Width: 2.9 mm (2.8–3.0). Abdomen: 2.6 mm (3.1–4.0). Pedipalp: Femur 1.9 mm (1.5–2.1), Tibia 1.2 mm (1.6–2.2), Basitarsus 0.9 mm (0.7–1), Distitarsus 0.7 mm (0.6–0.7), Tarsal claw 0.5 mm (0.4–0.6).

#### Color pattern (in alcohol)

Chelicerae, pedipalps and carapace yellowish-brown. Legs light yellowish-brown. Abdomen pale yellow. Color of live animals unknown.

#### Genitalia

Female gonopod (Fig 4A) cushion-like, with small lateral thin projections pointing backwards, resembling claws; projections not sclerotized; atrium not covered by the lateral projections, and with several glandular openings.

**Fig 5:**
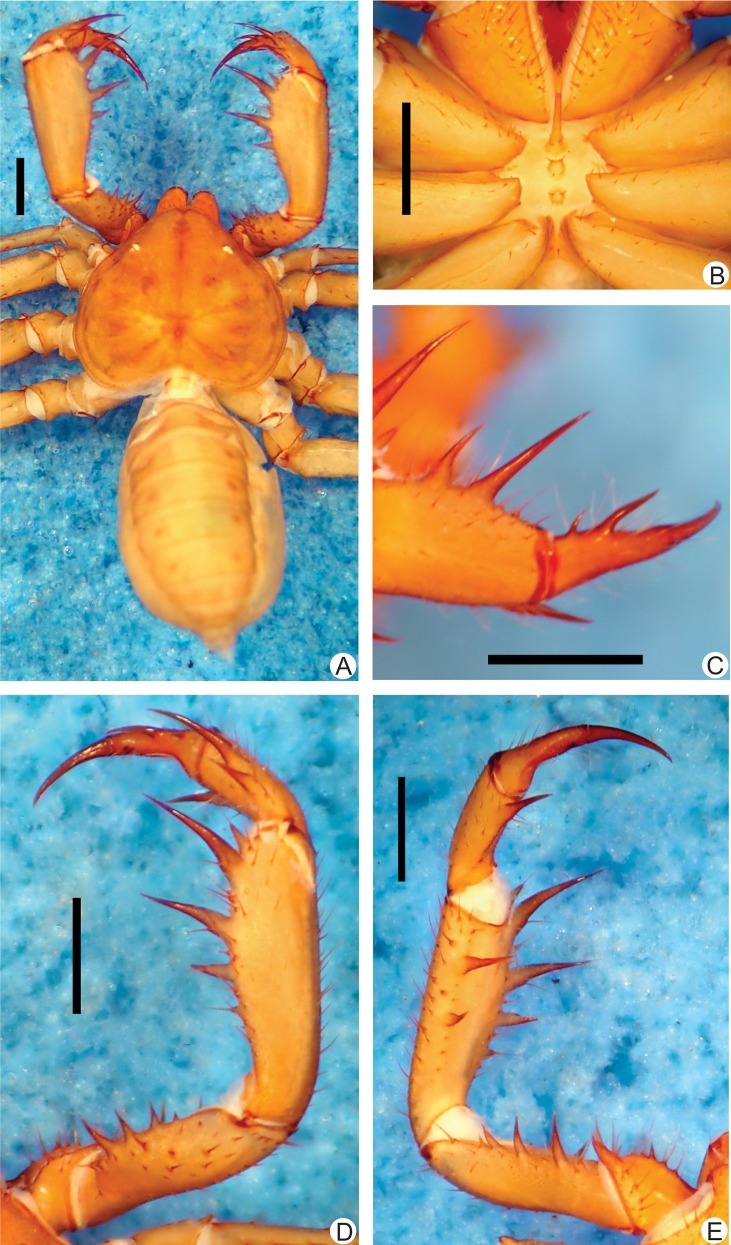
**A-E.** Habitus and details of the sternum and pedipalp of *Charinus ricardoi* sp. n. (female holotype, MZSP 22036) **A.** Dorsal habitus; **B.** Sternum; **C.** Frontal view of the right pedipalp basitiba, distitiba and claw; **D.** Dorsal view of the right pedipalp; **E.** Ventral view of the right pedipalp. Scale bars: 1mm.

***Charinus ricardoi* new species.** urn:lsid:zoobank.org:act:7AFD4D04-6BD2-4706-BFEE-432C366A49A5

(Figs 5A–5E, 2B and 3B)

#### Etymology

This species is named after Dr. Ricardo Pinto-da-Rocha (MZUSP, SP, BR), in recognition of his contribution to arachnology.

#### Type material

**Holotype**: BRAZIL: *Amazonas*: Presidente Figueiredo, Gruta Areia Branca (Areia Branca cave), 23-31.viii.2003, R. Pinto-da-Rocha *leg*. (1 Female, MZSP 22036). **Paratypes**: BRAZIL: *Amazonas*: Presidente Figueiredo, Gruta Areia Branca (Areia Branca cave) (3 females, MZSP 22036); Gruta dos Animais (Animais cave), 23-31.viii.2003, R. Pinto-da-Rocha *leg*. (1 juvenile, MZSP 22049; 1 female and 1 male, MZSP 22063).

#### Diagnosis

Absent median eyes and tubercle; weakly developed and pale lateral eyes; small and rounded meta and mesosternum; small basal spine of pedipalp distitarsus, ¼ the distal spine length; pedipalp almost perpendicular to the body, similar to that of *Paracharon caecus* Hansen, 1921; basitibia of leg IV divided in two pseudo articles; trichobothria of basitibia IV (*bt*) on the proximal third of the article; distitibia IV with 16 trichobothria; equidistant basal trichobothriae of distitibia IV (*bf*, *bc* and *sbf*); yellowish-brown body color; cushion-like gonopods without projections and with internal seminal receptacles; gonopods very similar to that of *Charinus bonaldoi* sp. n. (described below), but the pedipalp proportions and the size of the pedipalp articles are larger in *C*. *ricardoi* sp. n.

#### Description

**Carapace** (Fig 5A): flattened, wider than long, with an anterior depression in place of the absent median eye tubercle; from this depression starts a thin median furrow that reaches around the posterior area of the pair of lateral hump situated behind the lateral eye spots. Anterior margin with 5 to 7 small setae. Lateral eyes reduced to a small, whitish spot. Frontal process well developed, much longer than larger, with blunt, reborded apex.

#### Sternum (Fig 5B)

Same as *C*. *brescoviti* sp. n.

#### Abdomen (Fig 5A)

Same as *C*. *brescoviti* sp. n..

#### Chelicera

Cheliceral furrow (Fig 2B) with 4 internal teeth, the distal one short (almost the size of the second and third teeth) and bifid; the first cusp (1a) bigger than the second (1b). Fourth twice as long as the others and stouter. Teeth length: IV>Ia>Ib = II>III. Claw with 5 denticles, decreasing in size from the basal to the distal region.

#### Pedipalp

**Trochanter** (Fig 5D and 5E): large ventral apophysis, in the posterior border of the article, spiniform, bearing many strong setae, and with a blunt tip pointed forwards; 2 subequal spines, one on the medial third and the other on the distal tip of the prolateral face. **Femur** (Fig 5D and 5E): 3 dorsal spines decreasing in size from basal to distal; some specimens have a small fourth spine; each spine is 1/3 the size of the following (I>II>III); two prominent setiferous tubercle before the first spine; 4 ventral spines (I>II>III>IV) with similar sizes of the dorsal ones. **Tibia** (Fig 5D and 5E): dorsal main series with 3 spines (I>II>III); third is half the size of the second, and second is 2/3 the first; one accessory spine before the first, and no accessory spine after the third one, where is placed a small setiferous tubercle; 2 ventral spines, being the proximal half the distal. **Basitarsus** (Fig 5C, 5D and 5E): 2 dorsal spines, the basal half the size of the distal. 1 ventral spine at the distal half, 2/3 the size of the distal dorsal spine. **Distitarsus** (Fig 5C): 2 curved spines well developed, the distal half the size of the article and the basal 1/4 the size of the distal. Cleaning organ about ½ the article length. **Claw** (Fig 5C): long, with an acute, curved tip.

#### Legs

Same as *C*. *brescoviti* sp. n.. **Femur length** I>III>IV>II. Tibia I with 21 articles (one specimen with 23). Tarsus (basitarsus+distitarsus) I with 37 articles. **Leg IV**: **Basitibia:** divided into 2 pseudo-articles, with one trichobothrium in the middle of the proximal pseudo-article, and one trichobothrium in the base of the last pseudo-article. **Distitibia** (Fig 3B): 3 basal and 13 distal trichobothria (total of 16) trichobothrium *bc* mid-way to *bf* and *sbf*. **Basitibia-distitibia length** BT1>DT>BT3 = BT4>BT2. **Basitarsus**/**distitarsus ratio** 7/4, distitarsus tetramerous.

#### Measurements

**Female** (n = 3): Cephalothorax: Length: 2.61 mm (2.42–2.73), Width: 2.26 mm (2.67–3.80). Abdomen: 4.67 mm (4.4–5.0). Pedipalp: Femur: 2.30 mm (2.13–2.42), Tibia 2.29 mm (2.25–2.35), Basitarsus 2.20 mm (1.08–1.30), Distitarsus 0.87 mm (0.83–0.92), Tarsal claw 0.76 mm (0.71–0.85).

#### Color pattern (in alcohol)

Chelicerae, pedipalps and carapace yellowish. Legs lighter colored. Abdomen pale yellow. Live animals have similar color to the preserved ones.

#### Genitalia

Female gonopods (Fig 4B) cushion-like without lateral projections, and without sclerotized parts; atrium opened, with internal seminal receptacles, and several glandular openings; wall of the gonopods with an inflated aspect.

**Fig 6:**
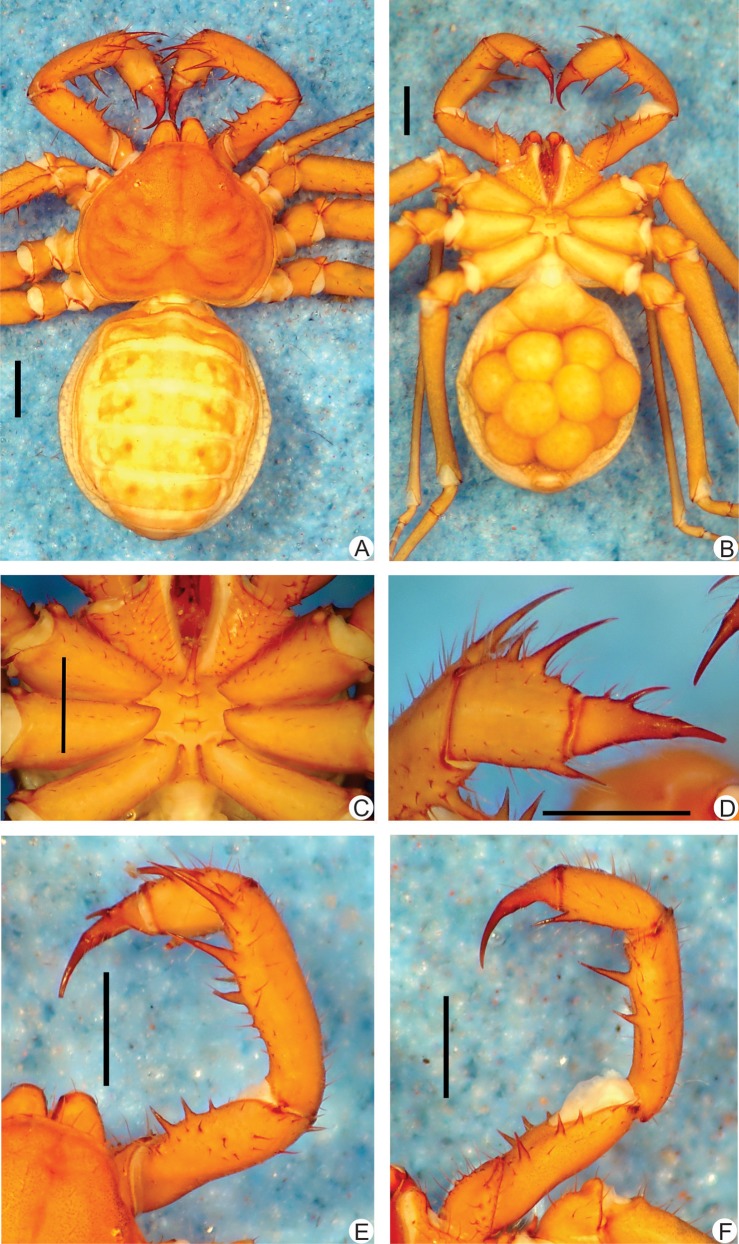
**A-F.** Habitus and details of the sternum and pedipalp of *Charinus bonaldoi* sp. n. (female holotype, MPEG AMB 061) **A.** Dorsal habitus; **B.** Ventral habitus of a female with eggs; **C.** Sternum; **D.** Frontal view of the right pedipalp basitiba, distitiba and claw; **E.** Dorsal view of the right pedipalp; **F.** Ventral view of the left pedipalp. Scale bars: 1mm.

***Charinus bonaldoi* new species.** urn:lsid:zoobank.org:act: 00826582-787B-481B-8F6E-0EE4786DE765

(Figs 6A–6F, 2C, 3C and 4C)

#### Etymology

This species is named after Dr. Alexandre Bragio Bonaldo (Museu Paraense Emílio Goeldi, PA, BR), in recognition of his contribution to arachnology.

#### Type material

**Holotype:** BRAZIL: *Pará*: Benevides, 18.i.2002, D.R.S., Souza & A. C. Souza (Female with eggs, MPEG AMB 061). **Paratypes:** BRAZIL: *Pará*: Benevides, 18.i.2002, D.R.S., Souza & A. C. Souza (5 Female, MPEG AMB 061); 06.xi.2001, D.R.S., Souza & A. C. Souza (1 Female and 3 juveniles, MPEG 061); 06.xi.2001, D.R.S., Souza & A. C. Souza (1 female, MPEG 060); 06.xi.2001, D.R.S., Souza & A. C. Souza (1 Female, MNRJ 09250).

#### Diagnosis

Absent median eyes and tubercle; weakly developed and pale lateral eyes; small and rounded meta and mesosternum; small basal spine of pedipalp distitarsus, ¼ the length of the distal; pedipalp almost perpendicular to body, similar to that of *C*. *ricardoi* sp. n. and *Paracharon caecus*; basitibia of leg IV divided in two pseudo articles; trichobothria of basitibia IV (*bt*) at the proximal third of the article; distitibia IV with 16 trichobothria; trichobothriae *bc* and *sbf* closer to each other than to *bf*; light brown body color; cushion-like gonopods without projections and with internal seminal receptacles.

#### Description

**Carapace** (Fig 6A): flattened, wider than long (ratio a little over 4/5), with a depression with two small setae in place of the absent median eye tubercle; from this depression starts a thin median furrow that reaches around the posterior area of the pair of lateral hump situated behind the lateral eye spots. Anterior margin with 5–7 small setae. Lateral eyes slightly reduced to a small, whitish spot. Frontal process well developed, much longer than larger, with blunt, reborded apex.

#### Sternum (Fig 6B and 6C)

Tri-segmented. Tritosternum with a round basis and projected anteriorly in a small blunt tubercle, with 2 apical, 2 median and 2 basal setae. Middle piece rounded, convex, with 2 setae and a few setulae; the setiferous tubercles are high, which gives an “M” shape to the middle piece. Third piece also rounded and convex, subequal to the middle piece and with two setae. Sternites separated from each other by the diameter of the middle piece.

#### Abdomen (Fig 6A and 6B)

Oblong, with almost indistinguishable punctuations, finer than in the carapace. Abdomen concave due to the presence of egg sac present.

#### Chelicera

Cheliceral furrow (Fig 2C) with 4 internal teeth, the distal bifid, the first cusp bigger than the second. Fourth twice as long the others and much stouter. Teeth length (from tip to basis) IV>Ia>Ib = II>III. Claw with 4 denticles, decreasing from the base to the distal part.

#### Pedipalp

**Trochanter** (Fig 6E and 6F): large ventral apophysis, at the posterior border of the article, spiniform, bearing strong setae and with a blunt tip pointed forwards, and 2 subequal spines, one at the median third and the other at the distal tip of the prolateral face. **Femur** (Fig 6E and 6F): 3 dorsal spines decreasing in size from basal to distal; the third spine half the second and three times smaller than last (I>II>III); two prominent setiferous tubercle before the first spine; 3 ventral spines (I>II>III) with similar sizes to the dorsal. **Tibia** (Fig 6E and 6F): main series with three spines (I>II>III); third half the size of the second, and second 2/3 the first; small accessory spine before the first spine and no small accessory spine after the third, instead a small setiferous tubercle is present; 2 ventral spines, the proximal half the size of the distal. **Basitarsus** (Fig 6D, 6E and 6F): 2 dorsal spines, the basal half the size of the distal. 1 ventral spine at the distal half, 2/3 the size of the distal dorsal spine. **Distitarsus** (Fig 6D): with 2 well developed curved spines, the distal half the size of the article, and the basal 1/3 the size of the distal. Cleaning organ about ½ the article length. **Claw** (Fig 6D): long, with an acute, curved tip.

#### Legs

**S**ame as *C*. *brescoviti* sp. n. **Femur length** I>III>IV>II. Tibia I with 21 articles. Tarsus (basitarsus+distitarsus) I with 37 articles. **Leg IV**: **Basitibia:** 2 pseudo-articles, with one basal trichobothrium at the last pseudo-article. **Distitibia** (Fig 3C): 3 basal and 13 distal trichobothria (total of 16); trichobothrium *bc* closer to *sbf* than to *bf*. **Basitibia-distitibia length** BT1>DT>BT3 = BT4>BT2. **Basitarsus**/**distitarsus ratio** 7/4, distitarsus tetramerous.

#### Measurements

**Females** (n = 4): Cephalothorax: Length: 2.58 mm (2.30–2.77), Width: 3.31 mm (2.78–3.70). Abdomen: 4.07 mm (3.70–4.42). Pedipalp: Femur 1.57 mm (1.2–1,77). Tibia 1.63 mm (1.30–2.14), Basitarsus 0.97 mm (0.89–1.24), Distitarsus 0.66 mm (0.54–0.84), Tarsal claw 0.57 mm (0.52–0.61).

#### Color Pattern (in alcohol)

Chelicerae, pedipalps and carapace yellowish. Legs lighter colored. Abdomen pale yellow. Live animals have color unknown.

#### Genitalia

Female gonopods (Fig 4C) cushion-like, without lateral projections, and without sclerotized parts; atrium opened, with internal seminal receptacles, and several glandular openings; wall of the gonopods with an inflated aspect; similar to the gonopod of *C*. *ricardoi* sp. n..

#### Natural history

Collected in the leaf litter.

**Fig 7:**
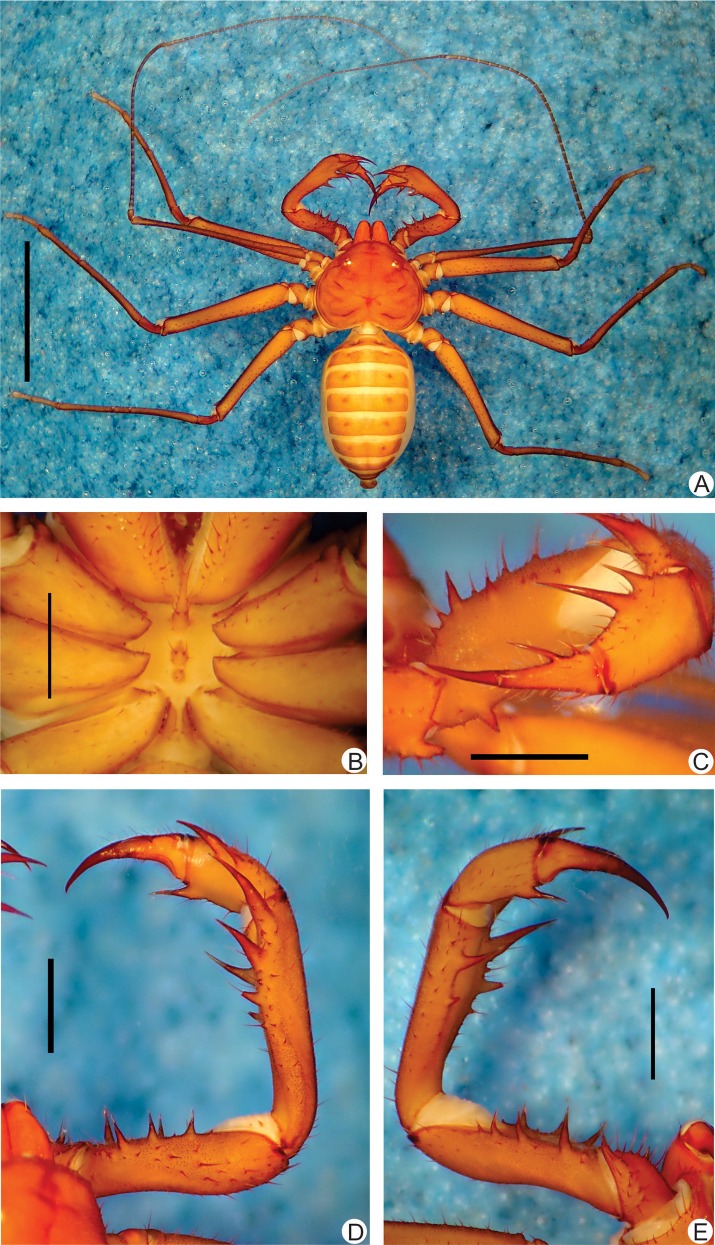
**A-E.** Habitus and details of the sternum and pedipalp of *Charinus guto* sp. n. (female holotype, MZSP 48146) **A.** Dorsal habitus; **B.** Sternum; **C.** Frontal view of the left pedipalp basitiba, distitiba and claw; **D.** Dorsal view of the right pedipalp; **E.** Ventral view of the right pedipalp. Scale bars: 1mm.

***Charinus guto* new species.** urn:lsid:zoobank.org:act: 8E381891-5040-4040-BA37-2BB0A067A10F

(Figs 7A–7E, 2D, 3D and 4D)

#### Etymology

This species is named after the arachnologist José Augusto Pereira Barreiros, nicknamed Guto (*in memoriam*), who collected some of the specimens of the type series.

#### Type material

**Holotype:** BRAZIL: *Pará*: Belém, Bosque Rodrigues Alves, 27.x.2002, R. Pinto-da-Rocha *leg*. (female, MZSP 48146). **Paratypes:** BRAZIL: *Pará*: Belém, Bosque Rodrigues Alves, 27.x.2002, R. Pinto-da-Rocha *leg*. (3 females, MZSP 48146); 27.x.2002, R. Pinto-da-Rocha *leg*. (1 female, MNRJ 09188); 08-XI-2001, J.A.P Barreirros (1 female juvenile, MPEG 059); 08.xi.2001, J.A.P Barreirros (1 female, MNRJ 09202); 08.iv.2001, A.B. Bonaldo *et al*. *leg*. (1 female, MPEG 037); 08.iv.2001, A.B. Bonaldo *et al*. *leg*. (1 female, MPEG 038).

#### Diagnosis

Absent median eyes and tubercle; well-developed lateral eyes, but pale; small and rounded meta and mesosternum; small basal spine of the distitarsus of the pedipalp, ¼ the length of the distal; tibia I with 21 articles in the and tarsus with 37; basitibia of leg IV divided in three pseudo articles; trichobothria of the basitibia IV (*bt*) at the proximal third of the article; distitibia IV with 14 trichobothria; equidistant basal trichobothriae of distitibia IV (*bf*, *bc* and *sbf*); light brown body color; cushion-like gonopods with lateral projections directed backwards covering the aperture of the internal seminal receptacles.

#### Description

**Carapace** (Fig 7A): flattened, wider than long, with an anterior depression in place of the absent median eye tubercle, from which starts a thin median furrow that reaches around the posterior area of the pair of lateral hump, situated behind the lateral eye spots. Anterior margin with 6 small setae. Many tiny punctuations, more abundant in the frontal area. Punctuations arranged in lines and spots, irradiating from the fovea and interspersed with glabrous areas. Three pairs of deep furrows, and a very rectangular deep fovea. First pair of furrows just behind the lateral boss not reaching the middle line. 4 lateral pairs of depressions (first one placed over the 1^st^ pair of furrows). Lateral eyes well developed. Frontal process well developed, much longer than larger, with blunt, reborded apex.

#### Sternum (Fig 7B)

Same as *C*. *brescoviti* sp. n. **Abdomen** (Fig 7A): same as *C*. *brescoviti* sp. n.

#### Chelicera (Fig 2D)

Cheliceral furrow with 4 internal teeth, the distal one bifid, the first cusp bigger than the second one. Fourth twice as long as the others and much stouter. Teeth length: IV>Ia>Ib = II>III. Claw with 5 denticles, decreasing in size.

#### Pedipalp

**Trochanter** (Fig 7D and 7E): ventral apophysis large, at the posterior border of the article, spiniform, bearing many strong setae, with a blunt tip pointed forwards, and 2 subequal spines, one at the median third and the other at the distal tip of the prolateral face. **Femur** (Fig 7D and 7E): 3 dorsal spines decreasing in size from proximal to distal; two small setiferous tubercle before spine I; 3 ventral spines (some specimens with 4), same relation of size as the dorsal, slightly larger than the dorsal. **Tibia** (Fig 7D and 7E): 3 dorsal spines (III>II>I). Spine II 1/3 spine I; spine III 2/3 II. 1 small setiferous tubercle closes to spine I, and one after spine III. 2 ventral spines, basal 1/3 the distal. **Basitarsus** (Fig 7C, 7D and 7E): 2 dorsal spines, basal ½ the distal. 1 ventral spine at the distal half, slightly bigger than the basal dorsal. **Distitarsus** (Fig 7C): long, with 2 curved spines, basal ½ distal. Cleaning organ about ½ the article length. **Claw** (Fig 7C): long, with an acute, curved tip.

#### Legs

Same as *C*. *brescoviti* sp. n. **Femur length** I>III>IV>II. Tibia I with 21 articles. Tarsus (basitarsus+distitarsus) I with 37 articles. **Leg IV**: **Basitibia:** 3 pseudo-articles, one distal trichobothrium on the proximal pseudo-article, and 1 basal trichobothrium on the distal pseudo-article. **Distitibia** (Fig 3D): 3 basal and 13 distal trichobothria; *bc* is equidistant to *bf* and *sbf* (Fig 3D). **Basitibia-distitibia length** BT1>DT>BT3 = BT4>BT2. **Basitarsus**/**distitarsus ratio** 7/4, distitarsus tetramerous.

#### Measurements

**Females** (n = 3): Cephalothorax: Length: 1.887 mm (1.73–2.13), Width: 2.47 mm (2.35–2.61). Abdomen: 4.06 mm (3.7–4.5). Pedipalp: Femur 1.13 mm (1.09–1.17), Tibia 1.09 mm (1.04–1.13), Basitarsus 0.68 mm (0.64–0.72), Distitarsus 0.45 mm (0.43–0.48), Tarsal claw 0.37 mm (0.36–0.38).

#### Color Pattern (in alcohol)

Chelicerae, pedipalps and carapace yellowish. Legs lighter colored. Abdomen pale yellow. Live animals with color pattern similar to the preserved specimens.

#### Genitalia (Fig 4D)

Female gonopods cushion-like, with lateral projections directed backwards, covering almost entirely the atrium opening; projections (claws) not sclerotized, wide, and with a blunt apex, as in *C*. *vulgaris* (see Miranda and Giupponi [7]).

#### Natural history

Collected in the leaf litter.

**Fig 8:**
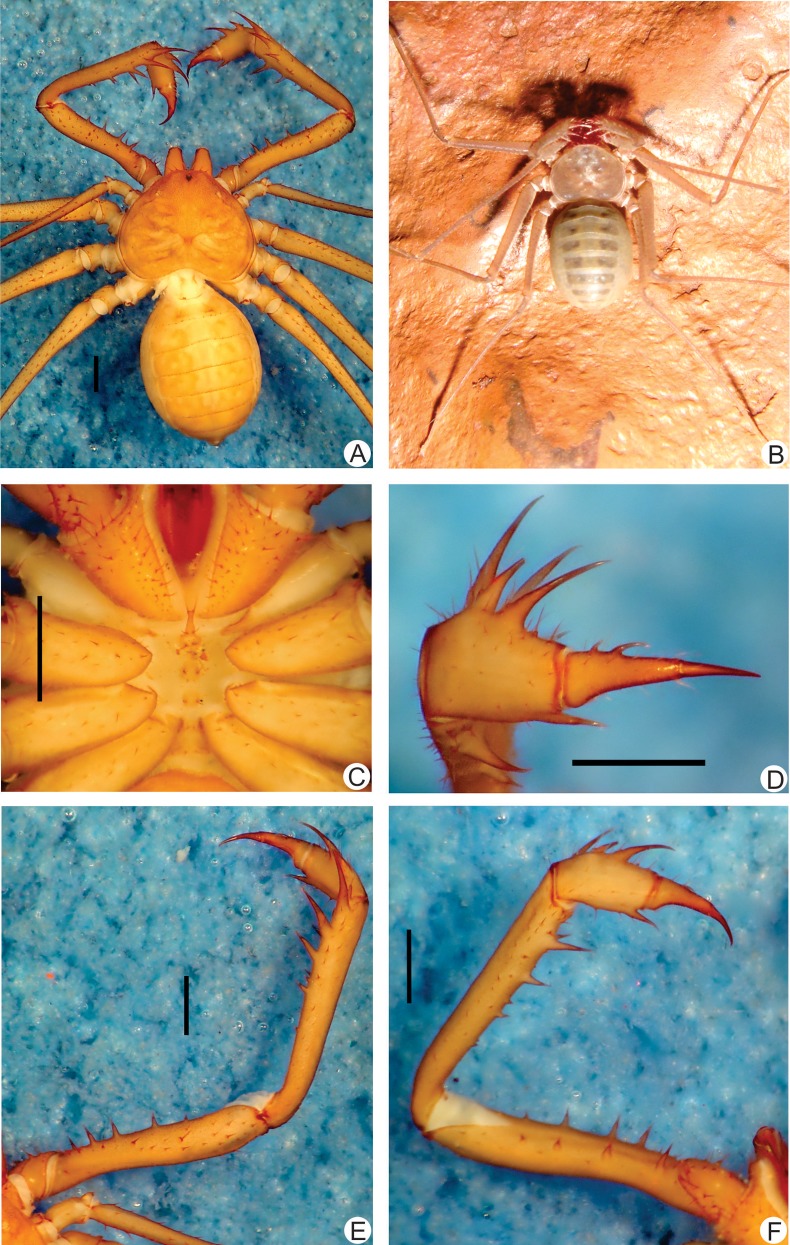
**A-F**. Habitus and details of the sternum and pedipalp of *Charinus carajas* sp. n. (male, MZSP 29126). **A.** Dorsal habitus; **B.** Live specimen in an iron cave in Carajás. **C.** Sternum; **D.** Frontal view of the right pedipalp basitiba, distitiba and claw; **E.** Dorsal view of the right pedipalp; **F.** Ventral view of the right pedipalp. Scale bars: 1mm. Photo B by Denis Pedroso.

***Charinus carajas* new species.** urn:lsid:zoobank.org:act: 630A7E0F-7D67-4929-A58F-EF5AC1B2E664

(Figs 8A–8F, 2E, 3E, 4E and 4G)

#### Etymology

This species is named after the mountain range where the species inhabits ("Serra Carajá", Caraja mountains). The name also refers to the important indigenous group called *karajas* or *iny mahãdu*, that occupy the region of the rivers Araguaia and Javaés in the states of Goiás, Mato Grosso, Tocantins and Pará, Brazil.

#### Material

**Holotype:** BRAZIL: *Pará*, Serra de Carajás, Gruta N4E-14 AF Flona Carajás, PA 592935mE / 9332514mN SAD’69, 7-12.x.2008 Andrade *et al*. *leg*. (1 female and 1 juvenile, MZSP 29136)

#### Paratype

BRAZIL: *Pará*, Serra de Carajás, FLONA de Carajás: Gruta N4E-22, 592107mE/9332976mN SAD’ 69, 7-12.x.2008 (1 male, MZSP 29126); Gruta N4E-95 59308mE/9332318mN SAD’ 69, 7-12.x.2008, Andrade *et al*. *leg*. (1 male, MZUSP 29127); Gruta N5S, 3-13.v.2005, Andrade & Arnoni *leg*. (1 juvenile, MZSP 28286); Gruta N4WS 15, 20.x.2006, Andrade *et al*. *leg*. (2 juveniles, MZSP 28292); Gruta N4E 33, 08-12.ii.2007, Andrade *et al*. *leg*. (2 juveniles, MZSP 28290); Gruta N5E 03 (nova 07), 22.iii2-03.iv, Andrade & Arnoni *leg*. (1 juvenile, MZSP 28281); Gruta N5S 01, 3-13.v.2005, Andrade & Arnoni *leg*. (1juvenile, MZSP 28285); Gruta N4E 21, 20.x-01.xi.2006, Andrade *et al*. *leg*. (1 juvenile, MZSP 28288); Gruta N4E 10 20.x-01.xi.2006, Andrade *et al*. *leg*. (1 juvenile, MZSP 28287); Gruta N5E 05 (nova 04), 22.iii-03.iv.2005, Andrade & Arnoni *leg*. (1 juvenile, MZSP 28283); Gruta N4E 22, 20.x-01.xi.2006, Andrade *et al*. col (2 juvenile, MZSP 28289); Gruta N5E 04 (nova 06), 22.iii-03.iv.2005, Andrade & Arnoni *leg*. (3 juvenile, MZSP 28282); *Parauapebas*, Projeto N5S, Morro I, Cav. GEM, 1782 (Est. Seca), 28.ii.2011,CARSTE *leg*. (1 male and 2 female, ISLA 3897); MII GEM 1722 EU (1 female, ISLA 3892), MII GEM 1755EU (1 male and 1 female, ISLA 3890); Gruta N4WS8 CL 589108mE/9326836mN SAD’ 69, 7-12.x-2008 (2 female, 1 male and 2 juvenile, MNRJ 09270, ex MZUSP 29132).

#### Addional material

Gruta N4E 61, FONA Carajás, PA, 08-12.ii.2007, Andrade *et al*. *leg*. (1 female and 3 juveniles, MZSP 28291); Gruta GEM15170, AF Tarzan, Carajás, PA 598042mE / 930054mN SAD’69 17-24.x.08, Andrade *et al leg*. (1 male and 3 juveniles, MZSP 29121); Gruta N5E 11 (nova 21), carajás, PA, 22.iii-03.iv.2005, Andrade & Arnoni *leg*. (1 female, MZSP 28284); Gruta GEM 1564 CL Tarzan, Carajás, PA, 599908mE/9301572mN SAD’69, 17-24.x.2008, Andrade *et al*. *leg*. (1 male, MZSP 29120); Gruta N4E-33 AF Flona Carajás, PA, 592939mE / 9332222mN SAD’69 7-12.x.2008, Andrade *et al*. *leg*. (1 male, MZSP 29125); Gruta N4WS8 AF Flona Carajás, PA 589108mE / 9326836mN SAD’69, 7-12.x.2008, Andrade *et al*. *leg*. (1 juvenile, MZSP 29137); Gruta N5S8 CL FLona Carajás, PA, 596003mE / 9325922mN SAD’69 7-12.x.2008, Andrade *et al*. *leg*. (1 male, MZSP 21131); Gruta N1-15, Mangangá; FLONA Carajás, PA, 28.ix-03.x.2007, Andrade *et al*. *leg*. (1 female, MZSP 29116); Gruta N1-15, mangangá, FLONA Carajás, PA, 28.ix-03.x.2007, Andrade *et al*. *leg*. (1 juvenile, MZSP 29115); Gruta N4E-08 CL, Flona Carajas, PA, 592957mE / 9332412mN SAD’69, 7-12.x.2008 Andrade *et al*. *leg*. (1 juvenile, MZSP 29134); Gruta N1-64 Amailton, FLONA Carajás, PA, 28.ix-03.x.2007, Andrade *et al*. *leg*. (1 female, MZSP 29114); Gruta N4E-61 AF Flona Carajás, PA, 592130mE / 9332403mN SAD’69 7-21.x.2008 Andrade *et al*. *leg*. (1 male, MZSP 29135); Gruta N4E-26 CL Flona Carajás, PA, 592154mE / 9332602mN SAD’69, 7-12.x.2008, Andrade *et al*. *leg*. (1 male, MZSP 29128); Gruta N4E-10 AF, Flona Carajás, PA 592889mE / 9332420mN SAD’69 7-12.x.2008, Andrade *et al*. *leg*. (1 juvenile, MZSP 29129); Gruta N4E-22 AF Flona Carajás, PA, 592107mE / 9332976mN SAD’69 7–12..x.2008 (1 juvenile, MZSP 29133); Gruta N1-25, Três Mosqueteiras, FLONA Carajás, PA, 28.ix-03.x.2007, Andrade *et al*. *leg*. (1 juvenile, MZSP 29113); Gruta n5S21 AF Flona Carajás, PA, 596716mE / 9327033mN SAD’69 7-12.x.2008, Andrade *et al*. *leg*. (1 juvenile, MZSP 29130); Gruta GEM 1590 CL Tarzan, Carajás, PA, 59891mE / 9301870mN SAD’69, 17-24.x.2008, Andrade *et al*. *leg*. (1 juvenile, MZSP 29123)

#### Diagnosis

Median and lateral eyes present, but median tubercle and lateral eyes strongly reduced; small and rounded meta and mesosternum; reduced tritosternun, slightly surpassing the base of the pedipalp coxae; dorsal femur with four spines; small basal spine of pedipalp distitarsus, ¼ the length of the distal; tibia I with 23 articles and tarsus with 42; basitibia of leg IV divided in three pseudo articles; trichobothria of basitibia IV (*bt*) at the proximal third of the article; distitibia IV with 16 trichobothria; equidistant basal trichobothriae of distitibia IV (*bf*, *bc* and *sbf*); pale yellow body color; cushion-like female gonopod with lateral projections directed backwards covering all the opening of the internal seminal receptacles (atrium); male gonopods with long, curved and wrinkled medial lobes; lateral lobes fimbriated; dorsal lobe surpassing the length of all other lobes and with elevated scales; secondary sexual dimorphism present, males with larger pedipalps, circa of two times the size of the female.

#### Description

**Carapace** (Fig 8A and 8B): flattened, wider than long; lateral and median eyes are reduced; median eye tubercle inside a trapezoid depression; individuals preserved in alcohol with a black spot under the median eyes tubercle. From the depression of the median eyes starts a thin median furrow that reaches around the posterior area of the pair of lateral hump situated behind the lateral eye spots. Anterior margin with 5 to 7 small setae. Frontal process well developed, much longer than larger, with blunt, rounded apex.

#### Sternum (Fig 8C)

Tri-segmented. Tritosternum with a round basis and projected anteriorly in a small blunt tubercle that reach the basis of the pedipalp coxa, with 2 apical, 2 median and 2 basal setae. Middle piece rounded, convex, with 2 setae and a few setulae. Third piece also rounded and convex, subequal to the middle piece, and with two setae. Sternites separated from each other by the diameter of the middle piece.

#### Abdomen (Fig 8A and 8B)

Same as *C*. *brescoviti* sp. n..

#### Chelicera (Fig 2E)

Cheliceral furrow with 4 internal teeth, the distal one bifid, the first cusp bigger than the second one. Fourth twice as long than the others and much stouter. Teeth length (from tip to basis) IV>Ia>Ib>III>II. Claw with 6 denticles, decreasing from the base to the distal part.

#### Pedipalp

**Trochanter** (Fig 8E and 8F): large distal, spiniform, ventral apophysis, bearing many strong setae and with a blunt tip pointed forwards, and 2 subequal spines, one at the median third and the other at the distal tip of the prolateral face. **Femur** (Fig 8E and 8F): 4 dorsal spines decreasing in size from basal to distal; first and second spines are subequal and the third is 2/3 the first (I>II>III); before the first spine, two prominent setiferous tubercle; 3 ventral spines (I>II>III) decreasing in size from proximal to distal; a fourth distal smaller spine is and a basal accessory spine before the first (slightly smaller than third) is present in some specimens. **Tibia** (Fig 8E and 8F): main series with 3 spines (I>II>III); in some specimens a small accessory spine after the third can be counted; third spine is 2/3 the second and the second is slightly smaller than the first; small accessory spine before the first spine; 3 ventral spines decreasing in size, the second and third 2/3 smaller than the following. **Basitarsus** (Fig 8D, 8E and 8F): 2 dorsal spines, the basal 2/3 the distal. 1 apical ventral spine, slightly smaller than the basal dorsal spine. **Distitarsus** (Fig 8D): with 2 well developed curved spines, the basal 1/3 the distal. Cleaning organ about ½ the article length. **Claw** (Fig 8D): long, with an acute, curved tip.

#### Legs

Same as *C*. *brescoviti* sp. n.. **Femur length** I>III>IV>II. Tibia I with 23 articles. Tarsus (basitarsus+distitarsus) I with 42 articles. **Leg IV**: **Basitibia:** 3 pseudo-articles, one trichobothrium at the limit of the basal and medial third of the basal pseudo-article, and another trichobothrium at the distal pseudo-article. **Distitibia** (Fig 3E): 3 basal and 13 distal trichobothria (total of 16); trichobothrium *bc* is little bit closer to *sbf* than to *bf*. **Basitibia-distitibia length** BT1>DT>BT3 = BT4>BT2. **Basitarsus**/**distitarsus ratio** 7/4, distitarsus tetramerous.

#### Measurements

**Males** (n = 2): Cephalothorax: Length: 2.49 mm (2.40–2.57), Width: 3.69 mm (3.6–3.79). Abdomen: 4.05 mm (3.65–4.46). Pedipalp: Femur 3.30 mm (2.61–4.0), Tibia 3.29 mm (2.72–3.85), Basitarsus 1.13 mm (0.84–1.43), Distitarsus 0.80 mm (0.74-.86), Tarsal claw 0.68 mm (0.65–0.71). **Females** (n = 1): Cephalothorax: Length: 2.38 mm, Width: 3.23 mm. Abdomen: 3.35 mm. Pedipalp: Femur 2.29 mm, Tibia 3.33 mm, Basitarsus 1.25 mm, Distitarsus 0.85 mm, Tarsal claw 0.75 mm.

#### Color Pattern (in alcohol)

Chelicerae, pedipalps and carapace yellowish. Legs lighter colored. Abdomen pale yellow. Color of live animals have are unknown.

#### Genitalia

**Female gonopods** (Fig 4E) cushion-like, with lateral projections directed backwards, covering completely the atrium opening; projections (claws) not sclerotized, wide, and with a rhombus apex, as in *C*. *guto* sp. n. and *C*. *vulgaris* (see Miranda & Giupponi, 2011). **Male gonopods** (Fig 4G) fistula with smooth tegument; medial lobes long, curved and wrinkled; median lamella integument wrinkled; dorsal lobe with erected projections, some acute and others straight (like a row of shark teeth); LoL2 fimbriated; LoL 1 covered with microvili; PI with large longitudinal folds.

#### Natural history

Inside cave, in a region called *canga* that contains iron ore.

**Fig 9:**
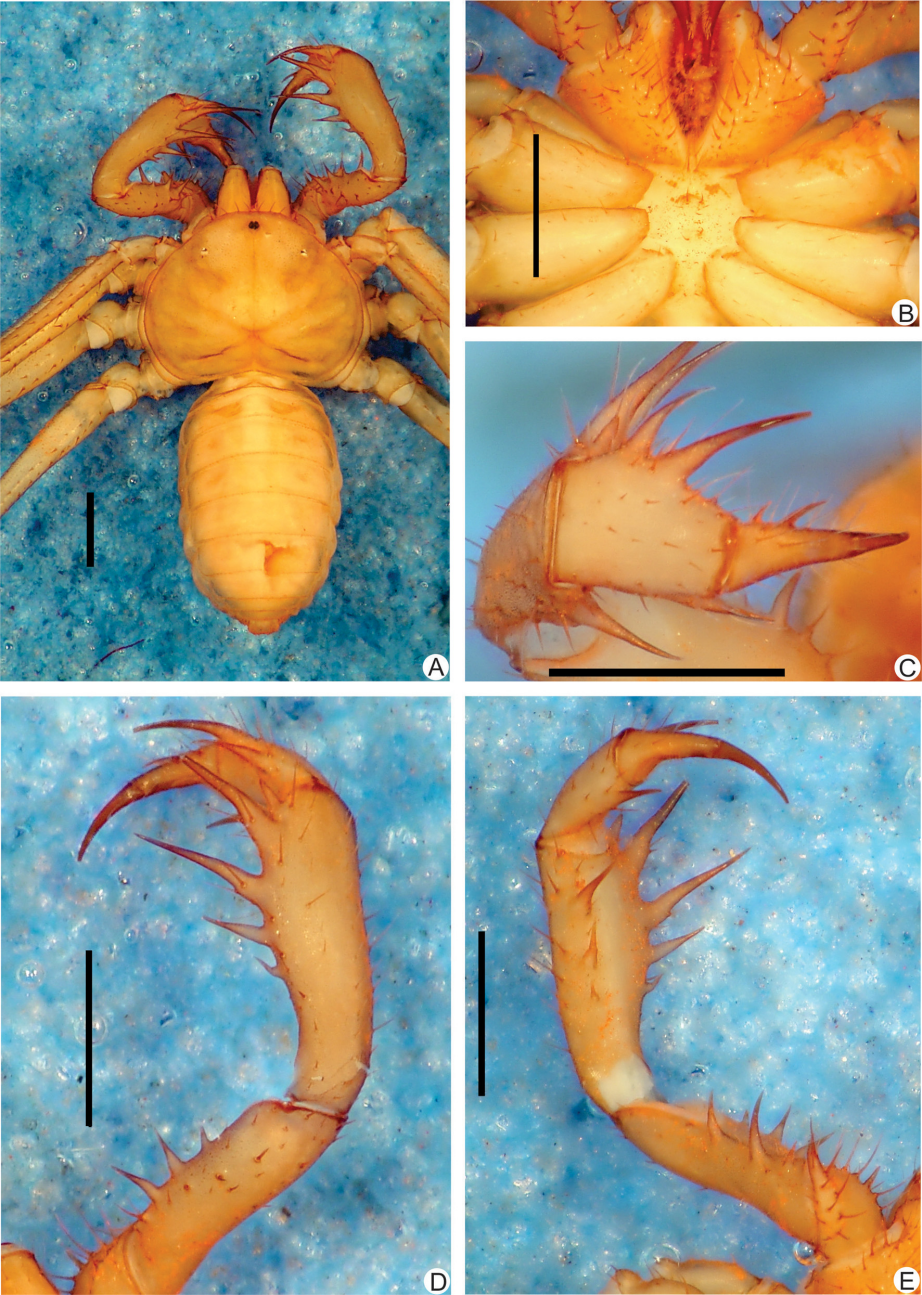
**A-E.** Habitus and details of the sternum and pedipalp of *Charinus orientalis* sp. n. (female paratype, MZSP 29118). **A.** Dorsal habitus; **B.** Sternum; **C.** Frontal view of the right pedipalp basitiba, distitiba and claw; **D.** Dorsal view of the right pedipalp; **E.** Ventral view of the right pedipalp. Scale bars: 1mm.

***Charinus orientalis* new species.** urn:lsid:zoobank.org:act: 9D4081AF-A043-4046-B206-801CE8010DB2

(Figs 9A–9E, 2F, 3F and 4F)

#### Etymology

The species name derives from the Latin *orientem*, which means east, referring to the name of the mountain range where the cave this species inhabits is located ("*Serra Leste*", east mountains).

#### Type material

**Holotype:** BRAZIL: *Pará*: Curionópolis, Projeto Serra Leste, Caverna SL-82 (Estação Seca), 07.vii.2010, Carste *leg*. (Female, MNRJ 09249). **Paratype:** BRAZIL: *Pará*: Serra do Leste, Flona de Carajás, 651411 mE / 9339212 mN SAD´69, 17-24/x/2008, Andrade *et al*. *leg*. (2 Females MZSP 29118); Curionópolis, Projeto Serra do Leste, Caverna SL-82 (Estação Seca), 07.vii.2010, Carste *leg*., (Female, MNRJ 09249); Curionópolis, Projeto Serra Leste, Caverna SL-82 (Estação Seca), 07.vii.2010, Carste *leg*. (3 Females and 2 Juveniles ISLA 3893); Flona Carajás, Gruta SL-75 CL, 651425 mE / 9340462 mN SAD´69, 17-24.x.08, Andrade *et al leg*. (Juvenile female, MZSP 29119); Flona Carajás, Gruta SL-82 CL, 650866 mE / 9341226 mN SAD´69 17-24.x.08 Andrade *et al leg*. (3 Females and 1 Juvenile female, MZSP 29124).

#### Diagnosis

Median and lateral eyes present, but strongly reduced median tubercle and lateral eyes (as in *C*. *carajas* sp. n.); median tubercle inside a depression; small and rounded meta and mesosternum; weakly sclerotized border of the sternum; pedipalp dorsal femur with three spines; basal spine of pedipalp distitarsus *circa* of ¼ the length of the distal; tibia I with 21 articles and tarsus I with 37; basitibia IV divided in three pseudo articles; trichobothria of basitibia IV (*bt*) at the proximal third of the article; distitibia IV with 16 trichobothria; equidistant basal trichobothriae of distitibia IV (*bf*, *bc* and *sbf*); pale yellow body color; cushion-like female gonopod with lateral projections directed backwards covering all the opening of the internal seminal receptacles (atrium).

#### Description

**Carapace** (Fig 9A): flattened, wider than long (ratio a little over 4/5) with an anterior depression with a slight elevation in its interior with two small setae (in place of the absent median eye tubercle). From this depression starts a thin median furrow that reaches around the posterior area of the pair of lateral hump situated behind the lateral eye spots. Anterior margin with 5 to 7 small setae. Median eyes absent. Lateral eyes slightly reduced to a small, whitish spot. Frontal process well developed, much longer than larger, with blunt, reborded apex.

#### Sternum (Fig 9B)

Tri-segmented. Tritosternum with a round basis and projected anteriorly in a small blunt tubercle, with 2 apical, 2 median and 2 basal setae. Middle piece rounded, convex, with 2 setae and a few setulae; the setiferous tubercles are elevated giving an “M” shape to the piece. Third piece also rounded and convex, subequal to the middle piece and with two setae. Sternites separated from each other by the diameter of the middle piece.

#### Abdomen (Fig 9A)

As in *C*. *brescoviti* sp. n.

#### Chelicera (Fig 2F)

Cheliceral furrow with 4 internal teeth, the distal one bifid, the first cusp bigger than the second. Fourth tooth twice as long as the others and much stouter. Teeth length (from tip to basis) IV>Ia>Ib = II>III. Claw with 4 denticles, decreasing from the base to the distal part.

#### Pedipalp

**Trochanter** (Fig 9D and 9E): large distal, spiniform, ventral apophysis, bearing many strong setae and with a blunt tip pointed forwards;2 subequal spines, one at the median third and the other at the distal tip of the prolateral face. **Femur** (Fig 9D and 9E): 3 dorsal spines decreasing in size from basal to distal; the third spine is half the second and three times smaller the first (I>II>III); before the first spine two prominent setiferous tubercle are present; 3 ventral spines (I>II>III) of similar sizes to the dorsal. **Tibia** (Fig 9D and 9E): main series with three spines (I>II>III); third half the size of the second and second 2/3 the first; small accessory spine before the first spine and no small accessory spine after the third spine, just of small setiferous tubercle is present; 2 ventral spines, the proximal half the distal. **Basitarsus** (Fig 9C): 2 dorsal spines, the basal half the size of the distal. 1 ventral spine at the distal half, 2/3 to the distal dorsal spine. **Distitarsus** (Fig 9C): with 2 curved well developed spines, the distal half the size of the article and the basal 1/3 the size of the distal. Cleaning organ about ½ the article length. **Claw** (Fig 9C): long, with an acute, curved tip.

#### Legs

Same as *C*. *brescoviti* sp. n. **Femur length** I>III>IV>II. Tibia I with 21 articles. Tarsus (basitarsus+distitarsus) I with 37 articles. **Leg IV**: **Basitibia:** 3 pseudo-articles, one basal trichobothrium at the last pseudo-article. **Distitibia** (Fig 3F): 3 basal and 13 distal trichobothria (total of 16). **Basitibia-distitibia length** BT1>DT>BT3 = BT4>BT2. **Basitarsus**/**distitarsus ratio** 7/4, distitarsus tetramerous.

#### Measurements

**Females** (n = 4): Cephalothorax: Length: 2.14 mm (1.87–2.43), Width: 2.92 mm (2.87–3,09). Abdomen: 3.10 mm (1.57–4.35). Pedipalp: Femur 1.73 mm (1.65–1.96), Tibia 1.72 mm (1.55–1.96), Basitarsus 0.82 mm (0.68–0.93), Distitarsus 0.65 mm (0.53–0.72), Tarsal claw 0.55 mm (0.52–0.58).

#### Color Pattern (in alcohol)

Chelicerae, pedipalps and carapace yellowish. Legs lighter colored. Abdomen pale yellow. Unknown color of live specimens.

#### Genitalia

**Female gonopods** (Fig 4F) cushion-like, atrium opening covered by the projections; projections (claws) not sclerotized, wide, and with a rhombus apex, as in *C*. *carajas* sp. n..

**Fig 10:**
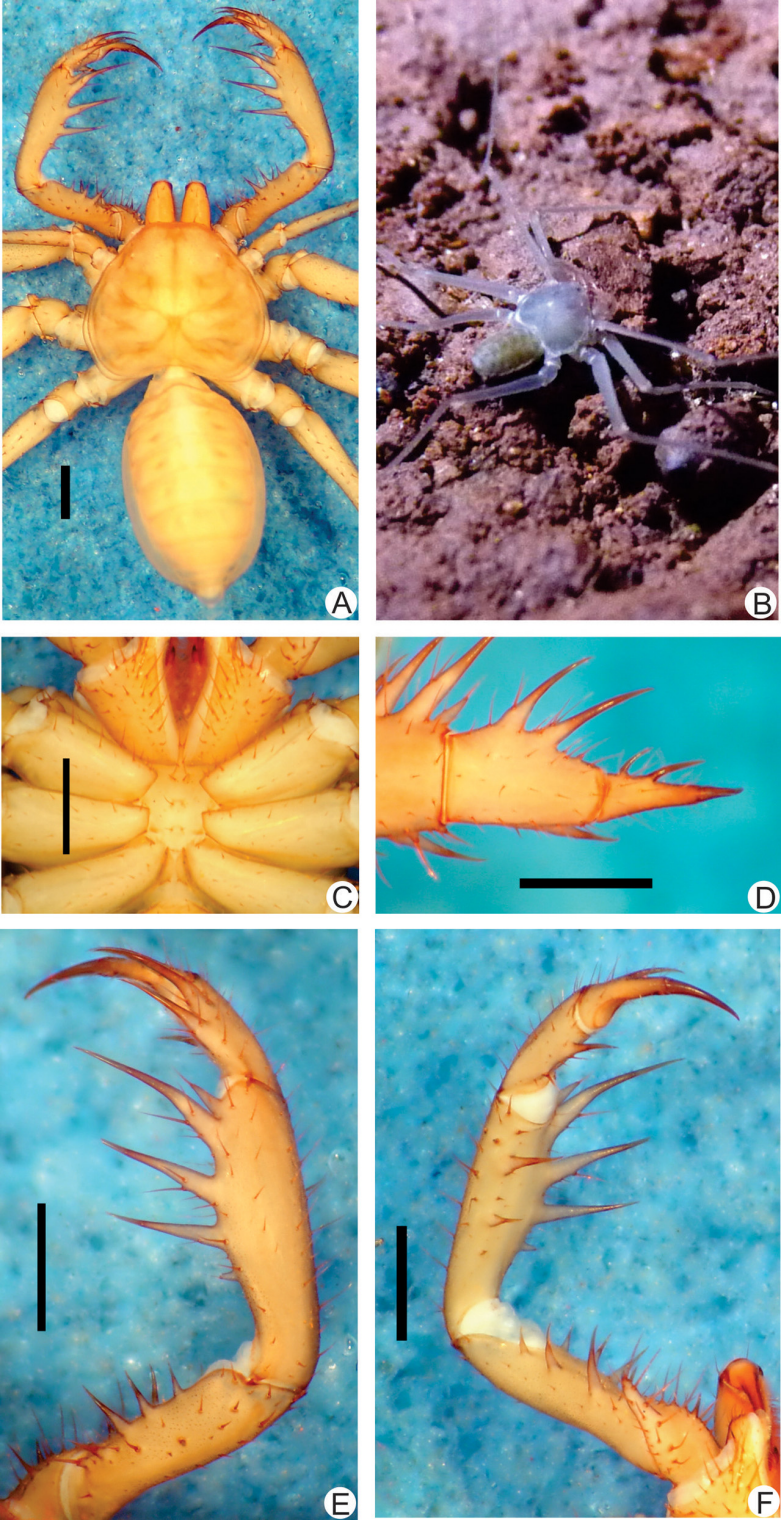
**A-F.** Habitus and details of the sternum and pedipalp of *Charinus ferreus* sp. n. (female holotype, MZSP 29104). **A.** Dorsal habitus; **B.** Live specimen in the soil of a cave in Carajás. **C.** Sternum; **D.** Frontal view of the right pedipalp basitiba, distitiba and claw; **E.** Dorsal view of the right pedipalp; F. Ventral view of the right pedipalp. Scale bars: 1mm. Photo B by Denis Pedroso.

***Charinus ferreus* new species.** urn:lsid:zoobank.org:act: 1F69E8D1-EDAB-488C-A831-DD5F9E7C2586

(Figs 10A–10F, 2G, 3G and 4H)

#### Etymology

The species name derives from Latin *ferrum*, referring to the iron ore cave from where this species were collected dwells.

#### Type Material

**Holotype:** BRAZIL: *Pará*: Serra de Carajás, FLONA de Carajás, Gruta S11 D64, 23.vii-02.ix.2007, Andrade *et al*. *leg*. (Female, MZSP 29104). **Paratype:** BRAZIL: *Pará*, Serra de Carajás, FLONA de Carajás, Gruta S11 D78, 23.viii.-02.ix.07, Andrade *et al*. *leg*., afotic. (1 female, MZUSP 29102); Gruta S11 A03, 23.viii.-02.ix.07, Andrade *et al*. *leg*., claro. (1 female, MZUSP 29103); Gruta S11 D64, 23.vii-02.ix.2007, Andrade *et al*. *leg*. (2 juvenile male and 2 juvenile female, MZSP 29104); Gruta S11 C27, 23.viii.-02.ix.07, Andrade *et al*. *leg*., claro. (2 juv, MZUSP 29105); Gruta S11 D39, 23.vii-02.ix.2007, Andrade *et al*. *leg*. (1 male and 1 juvenile, MZSP 29106); Gruta S11 D64, 23.viii.-02.ix.07, Andrade *et al*. *leg*., afotic. (1 female and 1 juvenile, MZUSP 29107); Gruta S11 B11, 23.viii.-02.ix.07, Andrade *et al*. *leg*., aphotic. (1 female, MZUSP 29108); Gruta S11 B11, 23.viii.-02.ix.07, Andrade *et al*. *leg*., claro. (1 juv, MZUSP 29109); Gruta S11 A05, 23.vii-02.ix.2007, Andrade *et al*. *leg*. (3 juvenile female, MZSP 29110). Gruta S11 D39, 23.viii.-02.ix.07, Andrade *et al*. *col*., claro. (1 female and 1 juv, MZUSP 29111); Gruta S11 A07, 23.viii.-02.ix.07, Andrade *et al*. *leg*., claro (1 juvenile, MZUSP 29112).

#### Diagnosis

Absent median eyes and tubercle; weakly developed and pale lateral eyes; small and rounded meta and mesosternum; reduced tritosternun, slightly surpassing the base of the pedipalp coxa; dorsal femur with three spines; subequal spines of pedipalp basitarsus; basal spine of pedipalp distitarsus large, circa of 2/3 the length of the distal; leg tibia I with 21 articles and tarsus I with 37; basitibia IV divided in three pseudo articles; trichobothria of the basitibia IV (*bt*) at the proximal third of the article; distitibia IV with 16 trichobothria; basal trichobothriae of distitibia IV *bc* and *sbf* closer to each other than to *bf*; pale yellow body color; male gonopod with long, curved and wrinkled medial lobes; lateral lobe fimbriated; median lobe surpassing the lateral and dorsal lobes.

#### Description

**Carapace** (Fig 10A): flattened, wider than long with an anterior depression in place of the absent median eye tubercle. From this depression starts a thin median furrow that reaches around the posterior area of the pair of lateral hump situated behind the lateral eye spots. Anterior margin with 6 small setae. Lateral eyes reduced to small rounded spots. Frontal process well developed, much longer than larger, with a rhombus apex.

#### Sternum (Fig 10C)

Tri-segmented. Tritosternum with a round basis and projected anteriorly in a small blunt tubercle, with 2 apical, 2 median and 2 basal setae. Midian and basal piece are reduced. Sternites separated from each other by the diameter of the middle piece.

#### Abdomen (Fig 10A)

Same as *C*. *brescoviti* sp. n.

#### Chelicera (Fig 2G)

Cheliceral furrow with 4 internal teeth, the distal bifid, the first cusp bigger than the second. Fourth tooth twice as long as the others and much stouter. Teeth length (from tip to basis) IV>Ia>Ib = II>III. Claw with 7 subequal rhombus denticles.

#### Pedipalp

**Trochanter** (Fig 10E and 10F): large distal, spiniform, ventral apophysis bearing many strong setae and with a blunt tip pointed forwards, and 2 subequal spines, one at the median third and the other at the distal tip of the prolateral face. **Femur** (Fig 10E and 10F): 3 dorsal spines decreasing in size from basal to distal (I>II>III); each 2/3 the size of the following; before the first spine two prominent setiferous tubercle, distant from each other, and at the same line of the main series of spines; 3 ventral spines (I>II>III) bigger than dorsal. **Tibia** (Fig 10E and 10F): main series with 3 spines (I>II>III); third 2/3 the second, which is 2/3 the first; small accessory spine before the first spine and one accessory spine after the third spine; 2 ventral spines, the proximal 2/3 the distal. **Basitarsus** (Fig 10D): 2 dorsal well developed spines, the distal the size of the article and the basal slightly smaller the distal. One ventral spine, at the distal half, 1/2 the basal dorsal spine. **Distitarsus** (Fig 10D): with 2 well developed curved spines, the distal bent; the basal 2/3 the distal. Cleaning organ about ½ the article length. **Claw** (Fig 10D): long, with an acute, curved tip.

#### Legs

Same as *C*. *brescoviti* sp. n. **Femur length** I>III>IV>II. Tibia I with 21 articles. Tarsus (basitarsus+distitarsus) I with 37 articles. **Leg IV**: **Basitibia:** 3 pseudo-articles, one medial trichobothrium at the last pseudo-article and one on the proximal pseudo-article. **Distitibia** (Fig 3G): 3 basal and 13 distal trichobothria (total of 16); two *bc* trichobothria, both closer to *sbf* than to *bf*. **Basitibia-distitibia length** BT1>DT>BT3 = BT4>BT2. **Basitarsus**/**distitarsus ratio** 7/4, distitarsus tetramerous.

#### Measurements

Male (n = 1): Cephalothorax: Length: 2.30 mm, Width: 3.04 mm. Abdomen: 3.74 mm. Pedipalp: Femur 1,87 mm, Tibia 1.83 mm, Basitarsus 1.04 mm, Distitarsus 0.70 mm, Tarsal claw 0.52 mm. **Females** (n = 1): Cephalothorax: Length: 2.54 mm, Width: 3.30 mm. Abdomen: 4.41 mm. Pedipalp: Femur 2.29 mm, Tibia 2.38 mm, Basitarsus 1.2 mm, Distitarsus 0.8 mm, Tarsal claw 0.65 mm.

#### Color Pattern (in alcohol)

Chelicerae, pedipalps and carapace pale yellow. Legs same as body. Abdomen pale yellow. Unknown color of live animals.

#### Genitalia

Male gonopods (Fig 4H):distal border of fistula smooth; integument of median lamella wrinkled; dorsal lobe with erected projections, with the acute or straight apex (resembling shark teeth); LoL2 fimbriated; LoL 1 covered with microvilli. PI surface with large longitudinal folds.

#### Natural History

Inside iron caves, in a region of Amazonia called “*canga*”.

#### Remarks

This species have troglomorphic characters, such as the almost complete absence of eyes.

**Fig 11:**
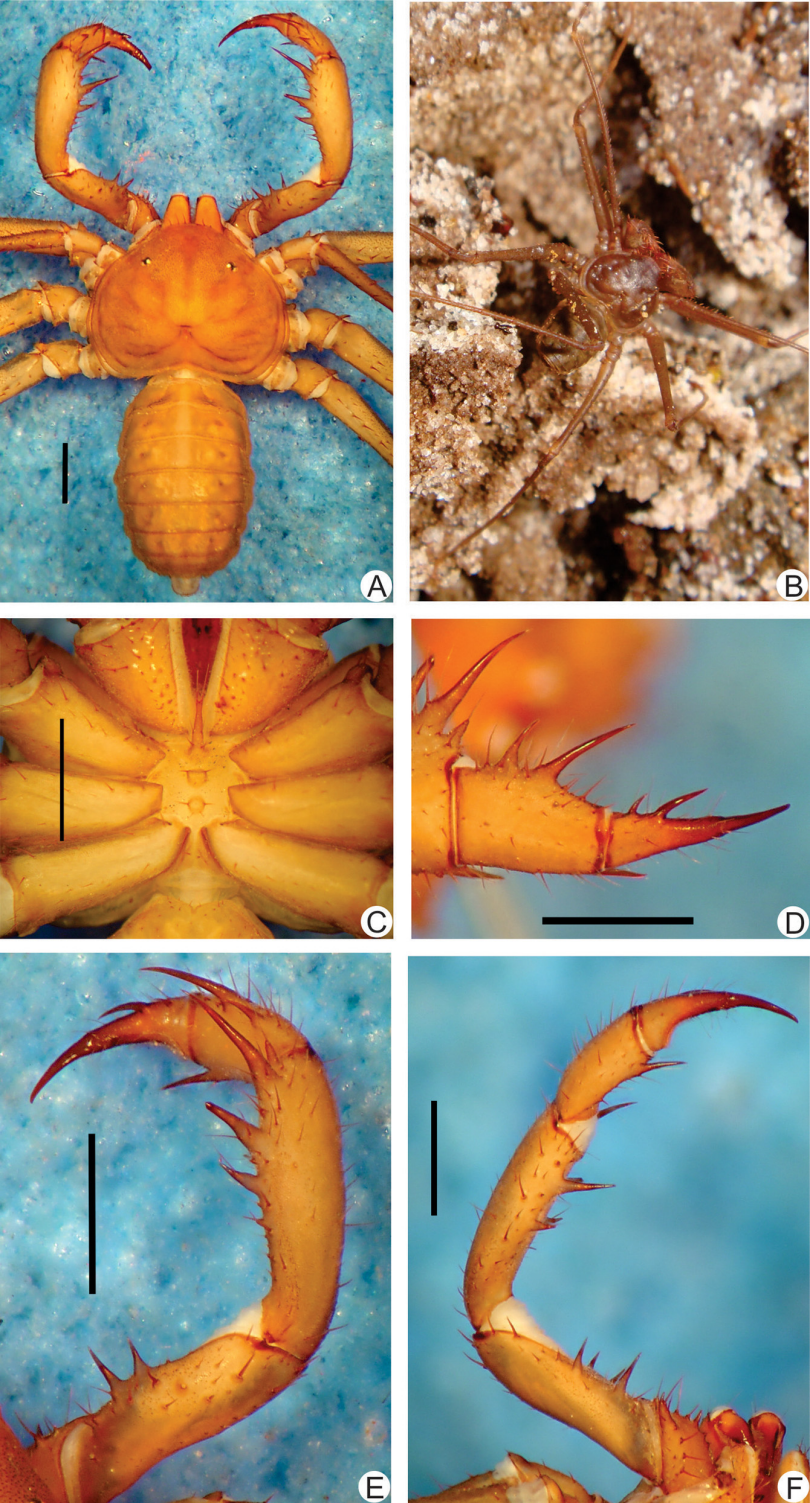
**A-F.** Habitus and details of the sternum and pedipalp of *Charinus bichuetteae* sp. n. (male holotype, MNRJ 09204). **A.** Dorsal habitus; **B.** Young live specimen in the soil in Altamira. **C.** Sternum; **D.** Frontal view of the right pedipalp basitiba, distitiba and claw; **E.** Dorsal view of the right pedipalp; F. Ventral view of the right pedipalp. Scale bars: 1mm. Photo B by Denis Pedroso.

***Charinus bichuetteae* new species.** urn:lsid:zoobank.org:act: 72D4630E-2DF3-49D2-AE7F-1ADEF14F6A5B

(Figs 11A–11F, 2H and 3H)

#### Etymology

This species is named after Dr. Maria Elina Bichuette (UFSCar, SP, BR), in recognition to her contribution to arachnology and cave biology.

#### Type material

**Holotype:** BRAZIL: *Pará*: Gruta do China, Vitória do Xingu, AL 44, 09.vii.2009, Pedroso, D., Pellegatti-Franco, Bichuette, M. E. & Scatolini, T. L. C. *leg*. (Male holotype, MNRJ 09204). **Paratypes**: BRAZIL: *Pará*: Vitória do Xingu, Gruta do China AL 44, 09.vii.2009, Pedroso, D., Pellegatti-Franco, Bichuette, M. E. & Scatolini, T. L. C. *leg*. (Male, MNRJ 09173); Altamira, Gruta do Sismógrafo, AL 05, 07.vii.2009, Pedroso, D., Pellegatti-Franco, Bichuette, M. E. & Scatolini, T. L. C. *leg*. (Female and female juvenile, MNRJ 09172); Vitória do Xingu, Paratizão, AL 19, 10.vii.2009, Pedroso, D., Pellegatti-Franco, Bichuette, M. E. & Scatolini, T. L. C. *leg*. (Female, MNRJ 09174); Altamira, B0103: Caverna Sugiro-Roncador, 2.i.2011, B0026: margem de rio, sob pedra, 3.iv.2011, B0049: Caverna Pedra da Cachoeira, 3.iv.2011, ME Bichuette, J.E. Gallão; D.M. von Schimonsky, DR Pedroso *leg*. (1 Male and 3 females, MNRJ 09311).

#### Diagnosis

Absent median eyes and tubercle; well-developed lateral eyes; small and rounded meta and mesosternum, strongly sclerotized; dorsal pedipalp femur with two spines; basal pedipalp distitarsus spine small, ¼ the length of the distal; Leg tibia I with 21 articles and tarsus with 37; basitibia IV divided in two pseudo articles; trichobothria of basitibia IV (*bt*) at the proximal third of the article; distitibia IV with 16 trichobothria; basal trichobothriae of distitibia IV *bc* and *sbf* closer to each other than to *bf*; pale yellow body color; cushion-like female gonopod with lateral projections directed backwards covering all the opening of the internal seminal receptacles (atrium).

#### Description

**Carapace** (Fig 11A): flattened, wider than long (ratio a little over 4/5) with an anterior smooth depression in place of the absent median eye tubercle. From this depression starts a thin median furrow that reaches around the posterior area of the pair of lateral hump, behind the lateral eye spots. Anterior margin with 6 small setae. Median eyes absent. Lateral eyes well developed. Frontal process projected downwards between the chelicerae with an blunt apex.

#### Sternum (Fig 11C)

Tri-segmented. Tritosternum with a round basis and projected anteriorly in a small blunt tubercle, with 2 apical, 2 median and 2 basal setae. Middle piece rounded, convex, with 2 setae and a few setulae; the setiferous tubercles are elevated giving an “M” shape to the piece. Third piece also rounded and convex, subequal to the middle piece and with two setae. Sternites separated from each other by the diameter of the middle piece.

#### Abdomen (Fig 11A)

Same as *C*. *brescoviti* sp. nov.

#### Chelicera (Fig 2H)

Cheliceral furrow with 4 internal teeth, the distal one bifid, the first cusp bigger than the second, and the distal slightly larger than half of the first. Teeth length (from tip to basis) I>II = III = IVa<IVb. Claw with 6 denticles, the two first are larger and the rest is subequal.

#### Pedipalp

**Trochanter** (Fig 11E and 11F): large distal, spiniform, ventral apophysis, bearing many strong setae and with a blunt tip pointed forwards with a slight curve, and 2 subequal ventral spines, one at the median third and the other at the distal tip; dorsally with one large setiferous tubercle. **Femur** (Fig 11E and 11F): 2 dorsal spines at the middle of the pedipalp, the basal larger than distal (I>II); before the first spine, two prominent setiferous tubercle; 3 ventral spines (I>II>III). **Tibia** (Fig 11E and 11F): main series with three spines (I>II>III); the second one third the first and the third two thirds the first; small accessory spine before the first spine and distally a small setiferous tubercle; 2 ventral spines, the basal one third of the first. **Basitarsus** (Fig 11D): 2 dorsal spines, the basal half the size of the distal. 1 ventral spine at the distal half, half the size of the dorsal. **Distitarsus** (Fig 11D): with 2 well developed curved spines, the basal one third the distal; distal spine of distitarsus slightly smaller than the distal spine of the basitarsus. Cleaning organ about ½ the article length. **Claw** (Fig 11D): long, with an acute, curved tip.

#### Legs

Same as *C*. *brescoviti* sp. n. **Femur length** I>III>IV>II. Tibia I with 21 articles. Tarsus (basitarsus+distitarsus) I with 37 articles. **Leg IV**: **Basitibia:** 2 pseudo-articles, one trichobothrium at the last pseudo-article at the edge of the basal third and median third. **Distitibia** (Fig 3H): 3 basal and 13 distal trichobothria (total of 16); *bc* closer to *sbf* than to *bf*. **Basitibia-distitibia length** BT1>DT>BT3 = BT4>BT2. **Basitarsus**/**distitarsus ratio** 7/4, distitarsus tetramerous.

#### Measurements

**Males** (n = 1): Cephalothorax: Length: 1,65 mm, Width: 2,17 mm. Abdomen: 1,26 mm. Pedipalp: Femur 1.04 mm, Tibia 1.00 mm, Basitarsus 0.62 mm, Distitarsus 0.49 mm, Tarsal claw 0.38 mm. **Females** (n = 3): Cephalothorax: Length: 2.27 mm (2.17–2.48), Width: 3.20 mm (3.00–3.352). Abdomen: 3.83 mm (3.26–4.04). Pedipalp: Femur 1.77 mm (1.57–1.87), Tibia 1.77 mm (1.61–1. 87), Basitarsus 1.0 mm (0.9–1.1), Distitarsus 0.7 mm (0.7–0.8), Tarsal claw 0.7 mm (0.6–0.7).

#### Color Pattern (in alcohol)

Chelicerae, pedipalps and carapace yellowish. Legs lighter colored. Abdomen pale yellow. Color of live have unknown.

#### Genitalia

**Male gonopods**: the male is a juvenile with the gonopods not well developed, so it is not described here as it can lead to misinterpretations. **Female gonopod**: cushion-like, without lateral projections, and with sclerotized parts (border of the atrium); the sclerotized region has small denticles; atrium open, with internal seminal receptacles; wall of the gonopods with an inflated aspect.

#### Natural history

The specimens collected were on the wall of the caves (Gruta do China, Gruta do Sismógrafo, Caverna Sugiro-Roncador, Caverna Pedra da Cachoeira).

### Identification key for the *Charinus* species from the Brazilian Amazonia (Fig 12)

1 Basitibia leg IV divided in 3 pseudoarticles...........................................2
1’ Basitibia leg IV divided em 2 pseudoarticles...................................6
2 Median eyes present....................................................................3
2’ Median eyes absent.............................................................4
3 Distitibia leg IV: trichobothria *bc* closer to *sbf* than to *bf* (Fig 3E)...........................................*C*. *carajas* sp. n.
3’ Distitibia leg IV: trichobothria *bc* equidistant to *bf* and *sbf* (Fig 3F)..................................*C*. *orientalis* sp. n.
4 Distitibia leg IV: trichobothria *bc* equidistant to *bf* and *sbf* (Fig 3D)........................................*C*. *guto* sp. n.
4’ Distitibia leg IV: trichobothria *bc* closer to *sbf* than to *bf* (Fig 3G)...........................................5
5 Large lateral eyes and oceli separated from each other...........................................*C*. *vulgaris*
5’ Reduced lateral eyes with oceli very close to each other (Fig 10A)..................................*C*. *ferreus* sp. n.
6 Median eyes present (Fig 1A)..................................*C*. *brescoviti* sp. n.
6’ Median eyes absent..................................7
7 Distitibia leg IV: trichobothria bc equidistant to bf and sbf (Fig 3B)..................................*C*. *ricardoi* sp. n.
7’ Distitibia leg IV: trichobothria bc closer to sbf..................................than to bf 8
8 Three dorsal and ventral spines on the pedipalp femur (Fig 6E and 6F)..................................*C*. *bonaldoi* sp. n.
8’ Two dorsal and ventral spines on the pedipalp femur (Fig 11E and 11F)..................................*C*. *bichuetteae* sp. n.

**Fig 12:**
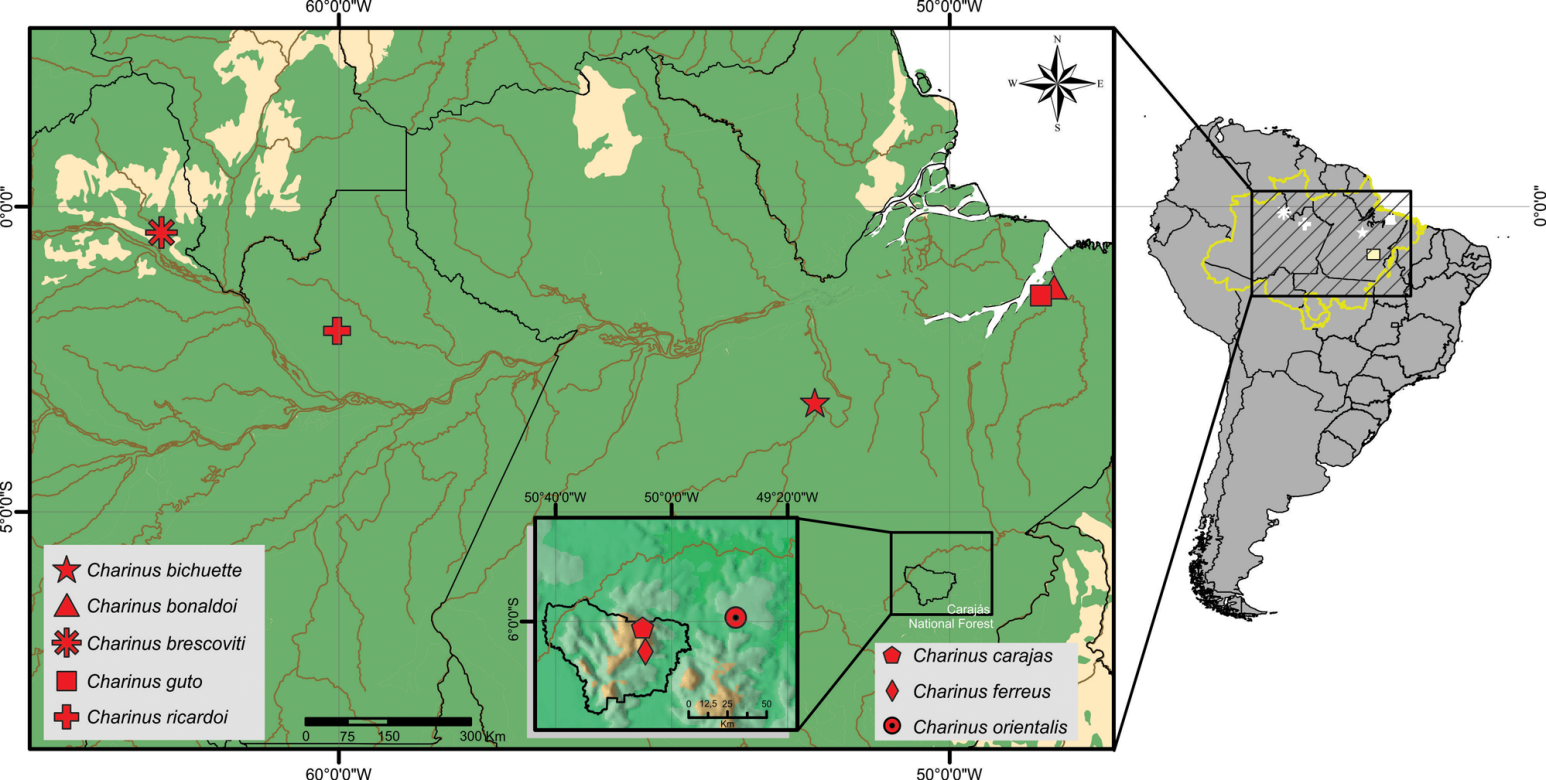
Distributional map of the new species. Green: ombrophilous forest. Yellow: white-sand forest (Amazonian Caatinga).

## Discussion

### Comparative morphology and taxonomy

The female genitalia of whip spiders is considered the most reliable structure to identify and determine species identity [2, 44]. Weygoldt [45] defined four species groups of *Charinus* based on this structure. They are: *Charinus australianus* species group, *Charinus brasilianus* species group, *Charinus bengalensis* species group, and *Charinus seychellarum* species group. The *australianus* group is the most widely distributed, with species in Australia, Brazil, Cuba, New Guinea, New Caledonia, and Peru (*C*. *australianus* (L. Koch, 1867), *C*. *acosta* (Quintero, 1983), *C*. *koepckei* Weygoldt, 1972, *C*. *milloti* Fage, 1939, *C*. *neocaledonicus* Simon, 1895 in Kraepelin, 1895, *C*. *pecki* Weygoldt, 2006, *C*. *pescotti* Dunn, 1949 and *C*. *vulgaris*) [7, 12, 46–52]. The group is recognized by the cushion-like gonopod and basitibia IV divided in three or four articles (Fig 3D).

All species here described also have a rounded cushion-like gonopod (Fig 4A–4H). Similarly to the only other Amazonian species of *Charinus* (*C*. *vulgaris*), none of the newly described species have basitibia IV divided in four articles. *C*. *brescoviti* sp. n., *C*. *ricardoi* sp. n., *C*. *bonaldoi* sp. n. and *C*. *bichuetteae* sp. n. have basitibia divided in two articles, and *C*. *guto* sp. n., *C*. *orientalis* sp. n. and *C*. *ferreus* sp. n. have basitibia IV divided in three articles (Fig 3D).

All Caribbean and one of the north South American *Charinus* species with described gonopod also have rounded cushion-like gonopods, and should be included in the *C*. *australianus* species group; they are *C*. *cubensis* (Quintero, 1983), *C*. *perezassoi* Armas, 2010, *C*. *platnicki* (Quintero, 1986), and *C*. *victori* Armas, 2010 [3, 53]. This way, the number of species in the *australianus* species group increases to 22, and this number can get higher once more gonopods of the known and new species are described.

Another feature of the Amazonian species of *Charinus* is the small size of their structures and appendages. All species known so far from Amazonia are smaller than those of the western part of South America (*C*. *brasilianus*, *C*. *montanus*, *C*. *asturius*, *C*. *acaraje*, *C*. *mysticus*, *C*. *troglobius*, *C*. *eleonore*, *C*. *potiguar* and *C*. *jiboassu*). Besides that, the Amazonian species have lower number of teeth on the chelicerae claw (*Charinus potiguar*, *C*. *eleonorae* and *C*. *troglobius*, for example, have 11, 10 and 9 teeth, respectively; on the other hand *Charinus ferreus* has 7; *C*. *brescoviti*, *C*. *carajas* and *C*. *bichuetteae*, 6; *C*. *ricardoi* and *C*. *guto*, 5; and *C*. *bonaldoi*, *C*. *orientalis* and *C*. *vulgaris*, 4).

### Conservational status of *Charinus* in Brazil

#### Threats and protection

The Brazilian species of *Charinus* are considered endemic as the majority of them are only known from their type locality, even after investigation in nearby areas or in places with similar conditions of the type locality [14, 41, 54–57]. *C*. *mysticus* and *C*. *eleonorae*, for example, are recorded only from one cave or group of caves. Due to the endemicity of *Charinus* species, environmental disturbances have a direct effect on the species survivor. The first troglobious *Charinus* described in Brazil (*Charinus troglobious*) is threatened by the destruction or alteration of the physic conditions of the caves they inhabit; this species is already in the red list of the Brazilian Environmental Ministry (Ministério do Meio Ambiente, MMA) considered as endangered [58].

In recent meetings between researchers and Brazilian environmental Federal agencies, a document was formalized and will be used as basis to the new official Environmental Ministry list of threatened species. This new list will changes the status of *C*. *troglobius* from threatened to critically threatened, and will add *C*. *acaraje* and *C*. *mysticus* as species with deficient data, *C*. *potiguar* as vulnerable, *C*. *asturius* as endangered, and *C*. *eleonorae* as critically endangered.

Likewise, of the eight species here described, four are in regions of intense human exploitation or environmental modification; moreover, they are not known from other areas besides of the type locality and have small population size; for these reasons, these species should be considered to be included in the list of threatened species; they are: *C*. *bichuetteae* sp. n., *C*. *carajas* sp. n., *C*. *ferreus* sp. n., and *C*. *orientalis* sp. n.. *Charinus bichuetteae* sp. n. is only found in caves that will be influenced by the lake of the hydroelectric dam Belo Monte, Pará. Studies showed that the higher level of the water will have negative influences (biologically [37, 59] and socially [60, 61]) in the region, but these effects were not taken into account by the governmental agencies and the building of the reservoir was authorized. This way, caves such as *Kararaô*, *China*, *Paratizão*, *Sismógrafo* and others will be affected [62]. Also, specific studies to understand the impact of the environmental alterations on the species populations that inhabit the caves were not made. With the flooding, some caves will be completely submerged (such as *Assurini* shelter, *Abutre* shelter and *Gravura* shelter), and others will have its dynamic altered (such as *China* cave, *Kararaô* cave, *Kararaô* Shelter, *Paratizão* shelter and *Sismógrafo*/*Tatu* shelter) [62]. The habitat viability in sustaining these species will be severely compromised.

The effects of constructing dams in Amazonia go beyond threatens to invertebrate or cave animals. Since the Brazilian government Program for the Acceleration of Growth started in 2007, a series of environmental unsustainable infrastructure projects were and are being built in Amazonia. Some of these counted with non-specialist researchers studying the fauna and flora surveyed to write reports on environmental impacts, generating large criticism on the criteria adopted by the government to evaluate the impact of the projects [34, 63]. For that reason, consequences such as long-term extinction induction [64] and block of fish migration [65] by hydroelectric dams are unavoidable problems of powerplants built in the lowlands of Amazonia [34, 64, 65].

The same can be said for the mining exploitation in the country, where the laws are being relaxed and soon companies will be allowed to extract ore from inside of preserved areas [39]. This kind of practice directly affects the new species here described *Charinus carajas* sp. n., *C*. *ferreus* sp. n. and *C*. *orientalis* sp. n.. The region where these species inhabits is a hematite mining area and all three were collected inside iron caves in an area called Canga (Brazilian name to isolated inselbergs on tops of mountains in the states of Minas Gerais and Pará, associated with superficial iron crust, characterized by the presence of iron stone and ferricrete soils, similar to the banded iron formations (BIFs) in South-Western Australia; it is also characterized by high local and regional diversity [66, 67]), and lots of those caves are being destroyed by one of the largest mining companies of the planet. Therewith, the environment is suffering large alterations [68]. It is not known whether these species inhabits also outside the caves or in other caves, so the destruction of the Canga presets a real threaten to these species. Besides *Charinus*, there are two endemic microwhip scorpion (Palpigradi) species known from the region (*Leptokoenenia thalassophobica* Souza & Ferreira, 2013 and *Leptokoenenia pelada* Souza & Ferreira, 2013). They occur in the same cave system as *C*. *carajas* sp. n. and *C*. *orientalis* sp. n. (*Serra Norte* and *Serra Leste*), highlighting the importance of the preservation of those endemic-species host caves.

According to the Brazilian laws (law decree number 6640), caves that are type locality are considered as highly relevant to protect. This way, it is suggested that, within the legal parameters used by the Brazilian Environmental Ministry (MMA and ICMBio), four of the new species (*C*. *carajas* sp. n., *C*. *orientalis* sp. n., *C*. *bichuetteae* sp. n. and *C*. *ferreus* sp. n.) be elected and included as critically endangered in the list of species in extinction risk, and their habitats be protected.

Outside Para State, in the rest of the Amazon Forest (where the other species of Amazonian *Charinus* occur), the conservation of the Forest is also debatable. In the last two years (2013–2015) there was an increase of more than 90% of the deforestment in the region and comparing January of 2014 and 2015, a decrease of 70% in the forest cover was detected [69]. The deforestation affects not only life in Amazonia, but influence the whole continent, changing the climate of South America [70]. These data, thus, show an urgent need for an active protection measure of the Amazonian forest and its biodiversity, and the most logic way is to start with the type localities of (mainly the) endemic species.

#### Potential distribution of *Charinus*

Vasconcelos, Giupponi and Ferreira [14] performed an analysis of potential distribution of *Charinus* and argued that "*Charinus* has most of its species represented on islands such as the Antilles, Solomon Islands in the Southwest Pacific, Ilha Bela in Brazil, Saint Thomas and Prince and Seychelles in Africa, or in portions near the continental coastlines, as in Venezuela, Peru, Panama, on the eastern borders of the Mediterranean, and India". They also add that "this predisposition to occupy coastline portions and islands is also revealed in the *Charinus* potential distribution". However, a careful analysis of the localities where *Charinus* species occur shows that its distribution are almost always areas of easy access, such as highways, waterways, and populated and touristic places. This means that the current distribution of *Charinus* is biased by the collecting effort. Generally, specimens of this genus are casually found, being this probably the main reason why *Charinus* is not well represented in non-touristic places, such as central areas of Brazil and many other countries (with exception of Cuba) and continents (such as Africa). To efficiently collect specimens of *Charinus* it is needed a direct expedition or lucky. This way, the potential distribution found by Vasconcelos, Giupponi and Ferreira [14] may pass a biased impression of high diversity in some areas (such as the Southeast region of Brazil) and low diversity in others (e.g. Amazonia). The analysis shows, for example, that the probability of presence of *Charinus* in the Amazonian plain is as small as places where *Charinus* (or even Amblypygi) do not exist, such as south South America (Chile, Argentina and Uruguay). But, actually, the diversity of *Charinus* species in Amazonia is highest than thought and predicted, and now eight new species are known from that region.

The map presented by Vasconcelos, Giupponi and Ferreira [14] also shows the occurrence of *Charinus* in the coastal area of Santa Catarina and Rio Grande do Sul states (southern Brazil), a place with conditions not similar to any other place where *Charinus* occur, and without a real register of the genus (this is a well collected region). This spot in the map is present due to a misunderstanding generated by the homonymy and posterior change of name of two cities. Mello-Leitão [71] described *C*. *schirchii* from a city called "Therezopolis", which at that time named two cities in different states, Santa Catarina and Rio de Janeiro, but Mello-Leitão [71] did not mention from which state the specimens came from. Afterwards, Harvey [1] catalogued *C*. *schirchii* from "Theresópolis (now Queçaba), Santa Catarina", and Vasconcelos, Giupponi and Ferreira [14] followed this, generating the occurrence spot in the southern region of Brazil. But, it is possible to assure that the "Therezopolis” referred to by Mello-Leitão [71] was from the state of Rio de Janeiro, once the collector of the specimens, Dr. Paul F. Schirch, gathered considerable zoological material from Serra dos Órgãos, mainly in the municipality of Teresópolis (currently spelled differently from the time of Mello-Leitão [71]). The collection of Dr. Paul F. Schirch was bought by the National Museum (Museu Nacional do Rio de Janeiro, [72]) and was incorporated to the scientific collection afterwards [73].

Therefore, we conclude that potential distributional maps should be carefully created (with critical analysis of the records inserted) and carefully analyzed after published. With the speed of publication of new species, the addition of species from completely different regions or from different environments turns this kind of study rapidly outdated.
